# Biological motion perception in autism spectrum disorder: a meta-analysis

**DOI:** 10.1186/s13229-019-0299-8

**Published:** 2019-12-18

**Authors:** Greta Krasimirova Todorova, Rosalind Elizabeth Mcbean Hatton, Frank Earl Pollick

**Affiliations:** 1 University of Glasgow, 62 Hillhead Street, Glasgow, G12 8AD UK; 2 Bradford District Care NHS Foundation Trust, Bradford, UK

**Keywords:** Autism spectrum disorders, Biological motion, Meta-analysis, Age, Emotion recognition

## Abstract

**Background:**

Biological motion, namely the movement of others, conveys information that allows the identification of affective states and intentions. This makes it an important avenue of research in autism spectrum disorder where social functioning is one of the main areas of difficulty. We aimed to create a quantitative summary of previous findings and investigate potential factors, which could explain the variable results found in the literature investigating biological motion perception in autism.

**Methods:**

A search from five electronic databases yielded 52 papers eligible for a quantitative summarisation, including behavioural, eye-tracking, electroencephalography and functional magnetic resonance imaging studies.

**Results:**

Using a three-level random effects meta-analytic approach, we found that individuals with autism generally showed decreased performance in perception and interpretation of biological motion. Results additionally suggest decreased performance when higher order information, such as emotion, is required. Moreover, with the increase of age, the difference between autistic and neurotypical individuals decreases, with children showing the largest effect size overall.

**Conclusion:**

We highlight the need for methodological standards and clear distinctions between the age groups and paradigms utilised when trying to interpret differences between the two populations.

## Background

Biological motion (BM), namely the movement of other humans, conveys information that allows the identification of affective states and intentions [1–3]. BM processing specifically is the ability of individuals to detect, label and interpret human movement and to allocate certain emotional states to it. Thus, BM is an important component of social perception. Moreover, neurotypically developing (NT) individuals have been shown to be able to readily extract socially relevant information from sparse visual displays [1, 2]. Specifically, point-light displays (PLDs), which portray BM with points located only on the major joints, are readily recognised as depicting differing actions by NT [4].

Pavlova [2] argues that an inability to extract socially relevant information from BM could have damaging effects on social functioning. In fact, individuals with an intellectual disability have been shown to have no problem in identifying different types of motion [5, 6], whereas individuals with social functioning difficulties such as autism spectrum disorder (ASD) have shown reduced ability in extracting social information from BM [7]. Indeed, ASD’s main diagnostic characteristics include problems with social interaction and communication as well as repetitive and/or restrictive behaviours [8]. Thus, the social impairment in ASD can, to some extent, be readily related to a reduced ability to extract information from BM.

However, findings on BM in ASD tend to be mixed [7]. For example, some studies, which investigated the identification or recognition of actions from BM [9–12], did not find significant differences between NT and ASD individuals, whereas others have found differences between the two groups [13–15]. Simmons et al. [7] and McKay et al. [14] argue that this is because there is variability between ASD individuals. Several factors have been suggested to introduce this variability.

One of these potential factors is age. Specifically, on the one hand, it appears that research in children tends to consistently show an impairment in BM interpretation [5, 13, 16]. Whilst, on the other hand, research in adults does not find differences in performance in action perception and BM recognition [9–11].

Person characteristics such as sex and IQ have also been suggested to contribute to the variability of results. Specifically, IQ has been identified as a predictor of performance in some studies [17, 18] but not in others [9, 19, 20]. Furthermore, a recent meta-analysis by Van der Hallen et al. [21] looked at local vs. global paradigms, where individuals have to ignore the global context to be able to focus and perform a task on the specific parts or vice-a-versa. They observed greater differences when the proportion of females was higher. Hence, these demographic characteristics of the samples should be investigated as potential contributors to the variability in the findings.

The task at hand has also been considered as a contributing factor. Koldewyn et al. [22] argue that individuals with ASD are able to identify BM presented through simple PLDs from noise and classify them; however, it is the extraction of higher order information, such as emotional content, that shows the largest performance difference. In fact, although Hubert et al. [9] and Parron et al. [12] did not find differences between NT and ASD in action recognition, they found differences in emotion recognition from biological motion for adults and children. Additionally, Fridenson-Hayo et al. [23] found that in children, this difference in emotion recognition from BM is evident for both basic (e.g. happy, sad) and complex emotions (e.g. disappointed, proud) as well as being evident cross culturally (Britain, Sweden, Israel). Thus, both children and adults with ASD tend to be less sensitive to emotional content.

It has been suggested that eye-tracking research can inform our understanding of the social difficulties in ASD. A review and meta-analysis of eye-tracking studies showed that in ASD, attention to social versus non-social stimuli may be reduced [24]. The analysis also found that decreased attention might be given to the eyes and increased attention to the mouth and body compared to NT individuals. However, Chita-Tegmark [24] noted that the results were very mixed. This may have been because the authors tried to include a large number of studies and thus inevitably included a mixture of more than one type of stimuli, including faces, eyes and bodies. Specifically, bodies contain vital social information and are perceptually different from faces [25]. Thus, different processes may be involved when looking at these different stimuli. Nevertheless, even when looking at eye-tracking studies focusing only on biological motion, the same variability is observed. Namely, in preferential looking paradigms, children have shown reduced visual orientation to biological motion [5, 26, 27]. This difference between NT and ASD has not been found in adults [28]. In contrast, Fujisawa et al. [29] show that pre-school children tend to have a greater preference for upright than inverted BM, which was additionally greater than that in NT children. Hence, it is apparent that inconsistencies in eye-tracking studies also exist but cannot be simply explained by age as a driving factor.

One study argued that the mixed findings in the BM literature within ASD are due to ASD utilising different brain networks which develop later in life. Hence, McKay et al. [14] investigated BM perception between ASD and NT and found that the brain areas that communicate with each other in ASD are not the same as the ones found in NT. Specifically, functional magnetic resonance imaging (fMRI) studies tend to find reduced activation in ASD for areas such as the superior temporal sulcus, middle temporal gyrus and inferior parietal lobule. These are all areas that have been found to be related to the perception and interpretation of human motion and actions [30–32]. NT individuals, however, show connectivity within areas involved with action and human motion observation—such as the inferior and superior parietal lobules. On the other hand, individuals with autism have been found to have brain networks that involve connectivity with the fusiform, middle temporal and occipital gyri, which are all areas considered to be involved in more basic level motion perception rather than action recognition [14, 31].

Similarly, the mirror neuron network (MNN) has been implied to be related to social functioning as it is associated with observing and understanding the actions of others. Thus, Kaiser and Shiffrar [33] argue that the MNN could contribute to the impairments seen in ASD. Moreover, Villalobos et al. [34] have shown reduced functional connectivity in the prefrontal mirror neuron area in individuals with ASD. The MNN has mainly been investigated in imitation paradigms [35, 36] and indeed, dysfunctional activation has been identified in individuals with ASD. However, since the MNN is also involved in understanding others’ actions, its activation during simple action observation has also been investigated in ASD because understanding others’ actions is an integral part of social functioning. Most commonly, mu-suppression has been used to assess human mirror activity [37] and reduced mu-suppression has been found in ASD participants in comparison to NT individuals both when performing and observing BM [35, 38]. Thus, it appears that the impairment in the MNN could be another contributing factor to the social difficulty present in BM perception in ASD.

In order to help bring clarity to the field, there is a need for a quantitative review of the research done on BM perception in ASD. Previous literature reviews have already argued for reduced ability in interpreting social information from BM and about the diagnostic utility of biological motion in ASD [33, 39]. In one such attempt, Van der Hallen et al. [40] conducted a meta-analysis on global motion visual processing differences between individuals with ASD and neurotypically developing individuals in behavioural paradigms. They included 48 studies—28 looked at coherent movement processing from random dot kinematograms and 20 looked at biological motion detection or discrimination of BM from other types of motion (i.e. scrambled). Global motion processing in their context refers to being able to combine several moving stimuli into a coherent shape (i.e. PLDs) or to perceive a coherent direction of the motion of dots despite the existence of unrelated distractor noise. Van der Hallen et al. [40] found overall differences between ASD and NT individuals in global motion processing but did not find a specific effect for biological motion, rather an effect that indicated a general decreased performance in detecting or recognising global motion patterns in perception paradigms. Whilst Van der Hallen et al. [40] found no effect of potential moderators on group differences; they suggest that this may have been due to underpowered studies rather than there being no real effect. However, they did not include emotion processing paradigms and only compared PLDs and random dot kinematograms despite there being other forms of biological motion paradigms, such as animated humans and videos of humans. Another attempt at summarising the behavioural findings in the field was done by Federici and colleagues [41]. They focused on characteristics of PLDs, the levels of processing (first-order/direct/instrumental) and the manipulation of low level perceptual features in PLDs. They partially answer the question of the effect of the utilised paradigm, showing that when inferring intentions/actions/emotions is required in the task and when temporal manipulations are made to the stimuli, the effects are larger. Unfortunately, their meta-analysis did not focus on the characteristics of the autistic individuals, which, as seen above, have also been suggested to introduce variability in the findings. Finally, whilst Van der Hallen et al.’s [40] and Ferderici et al.’s [41] meta-analyses address the need for a summarisation and exploration of the variability in the results in the literature to a certain extent, their meta-analyses do not fully answer the questions about participant characteristics and their role in the existing findings.

To be able to understand what could drive potential behavioural differences, it is important to also review brain imaging literature for potential answers. There have been some previous attempts to summarise this literature. A meta-analysis on the fMRI investigation of ASD, which included studies on social perception in ASD, found differences between the ASD and NT groups in both basic social tasks such as face recognition and biological motion recognition, and in complex social tasks—i.e. emotion recognition [42]. However, within social perception, face perception was also included which limits the conclusions that can be made for the perception of only human movement. Similarly, a systematic review by Hamilton [43] tried to summarise the electroencephalogram (EEG) literature on MNN and autism in BM observation, reporting that experiments probing the relationship between MNN and ASD have produced very mixed results. However, Hamilton [43] does not provide a quantitative summary of the analysis, only a narrative one.

Since there are inconsistencies in previous findings, behavioural, eye-tracking and brain imaging evidence will be reviewed to identify whether there is substantial evidence for decreased measures of performance in perceiving and understanding BM in individuals on the autism spectrum. We choose to focus solely on biological motion perception as body movement presents qualitatively and perceptually different information from faces and eye-gaze [25]. Moreover, we want to minimise any inflation or deflation of the effect size of the difference between the two groups, which could be caused by the inclusion of faces and eye-gaze information, which in turn could limit the scope of interpretation. We include studies which have used videos of real humans performing movements, cartoons, which represent humans or human body parts (i.e. hands) (collectively termed full-light displays), and PLDs as described above. The inclusion of both behavioural and physiological measures will allow us to develop a comprehensive understanding of the differences between ASD and NT individuals. Where enough data were available (only in behavioural studies), we also investigate the effects of different contributing factors such as the age, sex and IQ of the participants, the quality of the studies and the effect different paradigms might have on the size and direction of the effect sizes.

## Methods

### Protocol

Before commencing this meta-analysis, an informal protocol was agreed by all authors based on PRISMA guidelines [44]. Following these guidelines, the protocol includes details about the methodology and the steps taken to collect and analyse the data, which were agreed prior to commencing this meta-analysis. Through discussions throughout the meta-analytic process and as problems arose, small changes were agreed upon by all authors, such as the exact analysis software, publication bias measures, age categories, etc. The changes are indicated within the protocol. The protocol is available upon request.

### Study selection

In order to identify eligible studies, we conducted a systematic literature search. The computerised search involved using the following electronic databases: Dissertations & Theses A&I (ProQuest), Dissertation & Theses: UK & Ireland (ProQuest), Web of Science, PsycINFO (EBSCOhost) and MEDLINE (OVID). The following search terms were used ‘autis*’, ‘biological motion’, ‘human motion’, ‘asd’, ‘asperger*’, ‘childhood schizophrenia’, ‘kanner*’, ‘pervasive development* disorder*’, ‘PDD-NOS’, ‘PDD*’, ‘PLD*’,’point-light display*’, “action observation*”, “action observation network*”, ‘AON’. The asterisk represents truncation, allowing the search to find items containing different endings of the term. Dissertations and Theses databases were searched in order to identify unpublished experiments in an attempt to minimise bias. The search was limited to results in English. Additional file 1 shows the search strategies used and the number of results the search returned. The search included a wide time span as no lower time criterion was imposed on the search engines allowing us to access the first available records. Results included records up to and including the first week of November 2017. A second search was done in May 2019 for any additional records, due to the substantial time that had passed from the initial search.

The following exclusion/inclusion criteria were then used when screening the remaining records’ abstracts and full text:

1. Published before week one of November 2017(search 1) and May 2019 (search 2)
2. Published primary empirical articles and theses with non-published results—excluding review articles, opinion pieces, correspondences, case studies, and meta-analyses
3. Participants in the sample must have an ASD diagnosis
4. Diagnosis must be confirmed through ADOS, ADI-R or a clinician

4.1 Added during review process: additional diagnostic measures such as the 3-Di, DISCO; those that are specific to Asperger’s disorder, for example the Gilliam Asperger Disorder Scale (GADS, as cited in Price et al. [45]), the Asperger Syndrome (and high functioning autism) Diagnostic Interview (ASDI as cited in Price et al. [45]) and the high-functioning Autism Spectrum Screening Questionnaire (ASSQ as cited in Price et al. [45]) were also accepted as confirmation of ASD diagnosis. Additionally, the Chinese/Japanese equivalents of tests were accepted as in Wang et al. [46] and Fujisawa et al. [29].

5. Study must contain fMRI, EEG, eye-tracking and/or behavioural designs
6. An ASD and NT control group must be present and compared
7. Although human biological motion includes face motion and eye-gaze, only papers involving human body movement were included to provide a more focused review. These include full-light displays and PLDs
8. When stimuli that aim to minimise the availability of structural cues (e.g. PLDs) were used, the stimuli must represent human form with a minimum of two points for PLDs
9. Studies that used videos of people or cartoons where the face was not obstructed were not included as faces could confound with the participants’ performance
10. Papers that focus on imitation of biological motion were not included
11. If papers focusing on imitation included a separate analysis of BM observation, solely the BM observation was included where possible
12. Similarly, if paradigms included additional stimuli, but performance on the BM paradigm was analysed and could be extracted separately from the other stimuli, only that analysis was included
13. Only papers that included t-statistics, descriptive statistics and/or effects sizes were included Data requests were made to authors, where eligible papers did not include the necessary data.

Two reviewers independently screened the titles, abstracts and full texts against the eligibility criteria. Disagreements were discussed and resolved by the two reviewers or by consultation with the third author. The final decisions on inclusion/ exclusion of the studies were compared between the two reviewers. Cohen’s Kappa at the first search was calculated which equated to 64.07%. However, since Cohen’s Kappa is sensitive to distribution inequality [47] and ~ 92% of the records were classified as false positives, the prevalence index (0.816) and the prevalence-adjusted kappa (PABAK) of inter-rater reliability were calculated (PABAK = 87.98% inter-rater reliability, absolute agreement = 93.99%). To minimise effort at the second search, inclusion/exclusion was compared at abstract level and then at full-text level (Abstract level: Kappa = 70.72%, PABAK = 80.33%; Full-text: Kappa = 69.57%, PABAK = 71.43%)

The references of included records were screened by hand, split between the two reviewers. Five further records were identified.

### Coding and data extraction

Coding of the studies was split between the first and second author. The studies were not double coded; however, the studies coded by the second author were double-checked by the first author. Papers were coded and data was extracted for the following variables:

1. Sample size for each group
2. Age: Mean and Standard deviation were extracted for both the NT and ASD groups and each group was post-hoc classified into one of three age groups—children (≤ 13), adolescents (> 13 and ≤ 19) and adult (> 19)
3. Full-Scale IQ: Mean and standard deviation were extracted for both the NT and ASD groups
4. Non-verbal IQ: Mean and standard deviation were extracted for both the NT and ASD groups
5. Sex ratio: the sex ratio for each group was extracted and transformed into the proportion of females present in the sample
6. Paradigm: the type of paradigm used was extracted and categorised as 1—Detection of biological motion in noise or in comparison to another stimulus (usually upside down or scrambled PLD) [11, 13, 45]; 2—Action and subjective states categorisation or recognition [15, 20, 46]; 3—Emotional states categorisation [19, 23, 48]; 4—Passive viewing (only relevant in fMRI, EEG and eye-tracking). What category each study falls in can be seen in Tables 1 and 2. Although we initially attempted to separate detection in noise from recognition in comparison to other stimuli, the authors later decided that both tasks would require a similar process of integrating low level information into a coherent human form to perform the task. Thus, to create balanced categories and conceptually cohesive categories, the two categories were combined.
7. Type of stimulus: the stimuli were grouped into two categories: 1—PLDs; 2—Full-light displays—videos of real people or animations

**Table 1:** Summary of studies

Author(s) (year)	ASD sample					NT sample					Paradigm	Measure	Stimuli	Duration(s)	*g*	var. *g*	SE (*g*)	WoE	SQA
	*N*	Age	Sex ratio	FSIQ	NVIQ	*N*	Age	Sex ratio	FSIQ	NVIQ									
Actis-Grosso et al. [49]	20	22.8	15/5	118.9	/	25	22.3	21/4	/	/	AR	Accuracy	PLD (13 strips)	3 (max 5 min)	− 0.2	0.09	0.04	8.5	0.71
Alaerts et al. [50]	12	12/2	13.8	/	100.3	16	13/3	14.2	/	106.7	ER	Accuracy	PLD (12 points)	3	0.78	0.15	0.07	8	0.73
	12	12/2	13.8	/	100.3	16	13/3	14.2	/	106.7	ER	RT	PLD (12 points)	3	4.62	0.52	0.14		
	15	15/0	21.7	107.9	105.6	15	15/0	23.3	114.8	109.1	ER	Accuracy	PLD (12 points)	3	4.93	0.53	0.13		
	15	15/0	21.7	107.9	105.6	15	15/0	23.3	114.8	109.1	ER	RT	PLD (12 points)	3	− 1.53	0.17	0.08		
Alaerts et al. [30]	15	21.7	15/0	107.9	105.6	15	23.3	15/0	114.8	109.1	D	*d′*	PLD (12 points)	4	0.66	0.13	0.07	8.5	0.82
Annaz et al. [13]	23	8.83	/	/	/	34	8.25	/	/	/	D	*d′*	PLD (13 points)	1	1.15	0.08	0.04	8.5	0.8
	23	8.83	/	/	/	34	8.25	/	/	/	D	Thresholds	PLD (13 points)	1	0.89	0.08	0.04		
Atkinson [19]	13	30.9	12/1	106.2	105.2	16	26.7	14/2	106.6	108.4	ER	Accuracy	PLD (ns) and FLD	3	1.17	0.16	0.07	8.5	0.84
Binnerslеy [51]	14	12.917	14/0	/	/	15	/	/	/	/	D	*d'*	PLD (15 points)	1	0.22	0.13	0.07	8.5	0.8
Blake et al. [16]	12	/	/	/	/	9	8.417	/	/	/	D	*d'*	PLD	1	1.13	0.21	0.10	8	0.73
Cook et al. [52]	16	34.1	14/2	114.8	109	16	33.3	14/2	113	113	D	Thresholds	FLD	/	2.82	0.24	0.09	9	0.86
Couture et al. [53]	36	20.9	29/7	101.3	/	41	22.9	34/7	109.4	/	ER	Accuracy	PLD (12 points)	5–20	0.31	0.05	0.03	8	0.84
	36	20.9	29/7	101.3	/	41	22.9	34/7	109.4	/	ER	Accuracy	PLD (12 points)	5–20	0.75	0.05	0.03		
Cusack et al. [54]	15	16.09	15/0	103.1	/	15	15.54	15/0	104.8	/	AR	Thresholds	PLD (13 points)	2.5	0.18	0.13	0.07	8	0.82
	18	16.09	18/0	103.1	/	18	15.54	18/0	104.8	/	AR	Thresholds	PLD (13 points)	2.5	− 0	0.11	0.06		
	18	16.09	18/0	103.1	/	18	15.54	18/0	104.8	/	D	Thresholds	PLD (13 points)	1.5	0.3	0.11	0.06		
	15	16.09	15/00	103.1	/	15	15.54	15/00	104.8	/	D	Thresholds	PLD (13 points)	1.5	− 0.4	0.13	0.07		
	15	16.09	15/00	103.1	/	15	15.54	15/00	104.8	/	D	Thresholds	PLD (13 points)	2	− 0.4	0.13	0.07		
Edey et al. [55]	20	41.1	15/5	115.53	/	17	38.76	14/3	118.24	/	D	*d'*	FLD	/	− 0.05	0.1	0.05	8.5	0.91
	22	36.77	18/5	111.18	/	24	31.21	23/1	105.46	/	D	*d’*	FLD	/	− 0.26	0.08	0.04		
Freitag et al. [31]	15	17.5	13/2	101.2	93.3	15	18.6	13/2	112.1	106.8	D	Error rates	PLD	1.5	1.21	0.16	0.07	9	0.84
	15	17.5	13/2	101.2	93.3	15	18.6	13/2	112.1	106.8	D	RT	PLD	1.5	3.14	0.31	0.10		
Frideson-Hayo et al. [23]	20	7.45	18/2	/	/	22	7.5	19/3	/	/	ER	Accuracy	FLD	4–24	0.97	0.1	0.05	8.5	0.95
	20	7.45	18/2	/	/	22	7.5	19/3	/	/	ER	Accuracy	FLD	4–24	1.28	0.11	0.05		
	16	8.58	15/1	/	/	18	7.8	13/5	/	/	ER	Accuracy	FLD	4–24	1.15	0.13	0.06		
	16	8.58	15/1	/	/	18	7.8	13/5	/	/	ER	Accuracy	FLD	4–24	0.31	0.11	0.06		
	19	6.97	15/4	/	/	18	7.36	15/3	/	/	ER	Accuracy	FLD	4–24	0.41	0.11	0.05		
	19	6.97	15/4	/	/	18	7.36	15/3	/	/	ER	Accuracy	FLD	4–24	0.46	0.11	0.05		
Hubert et al. [9]	19	21.5	17/2	83.3	/	19	24.33	17/2	/	/	ER	Accuracy	PLD (5 & 10 points)	5	2.12	0.16	0.06	7.5	0.64
	19	21.5	17/2	83.3	/	19	24.33	17/2	/	/	AR	Accuracy	PLD (5 & 10 points)	5	0.85	0.11	0.05		
	19	21.5	17/2	83.3	/	19	24.33	17/2	/	/	AR	Accuracy	PLD (5 & 10 points)	5	1.8	0.14	0.06		
Jones et al. [17]	89	15.5	81/8	82.1	91.4	52	15.5	49/3	88.4	91.8	D	Thresholds	PLD (5 & 10 points)	5	0.3	0.03	0.01	8.5	0.77
Karuppali [56]	4	5.3	2/2	/	/	4	4.625	2/2	/	/	AR	Accuracy	PLD (15 points)	500–2000	3.25	1.04	0.36	7.5	0.69
Koldewyn et al. [22]	30	15.12	28/2	107.8	104.7	32	15.78	30/2	121.3	114.2	D	Thresholds	PLD (13 points)	2	0.82	0.07	0.03	8.5	0.82
	30	15.12	28/2	107.8	104.7	32	15.78	30/2	121.3	114.2	D	RT	PLD (13 points)	2	− 0.09	0.06	0.03		
Koldewyn et al. [57]	16	15.4	14/2	110.6	106.7	16	15.6	14/2	118.6	112.6	D	Thresholds (75%)	PLD (13 points)	2	1.01	0.13	0.06	8.5	0.86
Krakowski [58]	35	10.48	31/4	105.4	108.3	46	11.22	24/22	114	108.2	D	*d'*	PLD	/	0.6	0.05	0.02	8.5	0.84
Kroger et al. [59]	17	11.9	17/0	/	/	21	11.63	21/0	/	/	D	Accuracy	PLD (15 points)	1	− 0.2	0.1	0.05	9	0.86
	17	11.9	17/0	/	/	21	11.63	21/0	/	/	D	RT	PLD (15 points)	1	0.21	0.1	0.05		
Kruger et al. [60]	16	34.7	12/4	/	/	16	35	12/4	/	/	AR	Accuracy	PLD (13 points)	4	− 0.1	0.12	0.06	8	0.73
McKay et al. [14]	10	28.6	10/0	125	/	10	27.9	/	124.8	/	AR	Thresholds (50%)	PLD (15 points)	1	0.34	0.19	0.10	8.5	0.73
	10	28.6	10/0	125	/	10	27.9	/	124.8	/	AR	Thresholds (75%)	PLD (15 points)	1	0.8	0.2	0.10		
Morrison et al. [61]	106	24.28	95/11	108.9	/	95	24.17	84/11	116.28	/	D	*d'*	PLD	/	0.06	0.02	0.01	8.5	0.91
	106	24.28	92/11	108.9	/	95	24.17	84/11	116.28	/	ER	Accuracy	PLD	5–10	0.61	0.02	0.01		
Murphy et al. [10]	16	25.56	13/3	/	/	16	26.4	13/3	/	/	D	*d'*	PLD (11 points)	~ 6.5	0.23	0.12	0.06	8.5	0.84
	16	25.56	13/3	/	/	16	26.4	13/3	/	/	D	*d'*	PLD (11 points)	~ 6.5	0.32	0.12	0.06		
	16	25.56	13/3	/	/	16	26.4	13/3	/	/	D	*d'*	PLD (11 points)	~ 6.5	0.27	0.12	0.06		
	16	25.56	13/3	/	/	16	26.4	13/3	/	/	D	*d'*	PLD (11 points)	~ 6.5	0.46	0.12	0.06		
	16	25.56	13/3	/	/	16	26.4	13/3	/	/	D	*d'*	PLD (11 points)	~ 6.5	0.29	0.12	0.06		
	16	25.56	13/3	/	/	16	26.4	13/3	/	/	D	Error rates	PLD (11 points)	~ 6.5	0.53	0.12	0.06		
	16	25.56	13/3	/	/	16	26.4	13/3	/	/	D	Error rates	PLD (11 points)	~ 6.5	0.45	0.12	0.06		
	16	25.56	13/3	/	/	16	26.4	13/3	/	/	D	Error rates	PLD (11 points)	~ 6.5	0.37	0.12	0.06		
	16	25.56	13/3	/	/	16	26.4	13/3	/	/	D	Error rates	PLD (11 points)	~ 6.5	0.45	0.12	0.06		
	16	25.56	13/3	/	/	16	26.4	13/3	/	/	D	Error rates	PLD (11 points)	~ 6.5	0.19	0.12	0.06		
	16	25.56	13/3	/	/	16	26.4	13/3	/	/	D	RT	PLD (11 points)	~ 6.5	0.58	0.12	0.06		
	16	25.56	13/3	/	/	16	26.4	13/3	/	/	D	RT	PLD (11 points)	~ 6.5	0.46	0.12	0.06		
	16	25.56	13/3	/	/	16	26.4	13/3	/	/	D	RT	PLD (11 points)	~ 6.5	0.22	0.12	0.06		
	16	25.56	13/3	/	/	16	26.4	13/3	/	/	D	RT	PLD (11 points)	~ 6.5	0.29	0.12	0.06		
	16	25.56	13/3	/	/	16	26.4	13/3	/	/	D	RT	PLD (11 points)	~ 6.5	0.27	0.12	0.06		
Nackaerts et al. [15]	12	34.9	7/5	111.5	105.7	12	31.5	7/5	115.5	115.3	ER	Accuracy	PLD (12 points)	3	1.71	0.22	0.10	8	0.8
	12	34.9	7/5	111.5	105.7	12	31.5	7/5	115.5	115.3	D	*d'*	PLD (12 points)	3	1.28	0.19	0.09		
	12	34.9	7/5	111.5	105.7	12	31.5	7/5	115.5	115.3	D	Accuracy	PLD (12 points)	3	1.12	0.18	0.09		
Parron et al. [12]	23	11.583	20/3	93.7	89	23	12	20/3	/	/	ER	Accuracy	PLD (10 points)	~5	5.51	0.41	0.09	8.5	0.73
	23	11.583	20/3	93.7	89	23	12	20/3	/	/	AR	Accuracy	PLD (10 points)	~5	1.9	0.12	0.05		
	23	11.583	20/3	93.7	89	23	12	20/3	/	/	AR	Accuracy	PLD (10 points)	~5	3.08	0.19	0.06		
Philip et al. [48]	23	32.5	16/7	101.5	104.4	23	32.4	17/6	111.2	113.4	ER	Accuracy	FLD	5–10	1.6	0.11	0.05	9	0.84
Price et al. [45]	14	14.14	14/0	/	57.14	16	14.08	16/0	/	52.63	D	Accuracy	PLD	5	1.14	0.15	0.07	8.5	0.8
	14	14.14	14/0	/	57.14	16	14.08	16/0	/	52.63	D	*d'*	PLD	5	0.82	0.14	0.07		
	14	14.14	14/0	/	57.14	16	14.08	16/0	/	52.63	D	*d'*	PLD	5	0.64	0.13	0.07		
Saygin et al. [11]	16	33.75	13/3	112.2	107.2	20	37.75	14/7	113.2	108.6	D	Thresholds	PLD (12 points)	0.583	0.08	0.11	0.06	8	0.73
Sotoodeh et al .[62]	20	11.3	17/3	/	77.3	20	11.4	17/3	/	106	AR	Accuracy	PLD (13 points)	2s (3 times)	0.85	0.1	0.05	9	0.82
	20	11.3	17/3	/	77.3	20	11.4	17/3	/	106	AR	RT	PLD (13 points)	2s (3 times)	1.85	0.14	0.06		
Swettenham et al. [63]	13	9.583	/	/	/	13	8.5	/	/	/	AR	Accuracy	PLD (13 points)	/	0.39	0.15	0.08	8.5	0.77
Turi et al. [64]	19	11.49	16/3	105.9	/	18	11.94	14/4	107.6	/	AR	*d'*	PLD (23 points)	0.90 ± 0.15	1.64	0.14	0.06	9	0.8
	19	11.49	16/3	105.9	/	18	11.94	14/4	107.6	/	AR	*d'*	PLD (23 points)	0.90 ± 0.15	0.44	0.11	0.05		
van Boxtel et al. [20]	16	14.04	12/4	101.5	99.94	17	13.32	13/4	112.2	108.6	AR	Thresholds	PLD	1	0.27	0.12	0.06	9	0.82
von der Luhe et al. [65]	16	41.56	12/4	116.9	/	16	36.19	10/6	115.3	/	D	Accuracy	PLD	3.6–4.3	0.47	0.12	0.06	8.5	0.89
	16	41.56	12/4	116.9	/	16	36.19	10/6	115.3	/	D	Accuracy	PLD	3.6–4.3	0.53	0.12	0.06		
Wang et [46].	21	5.588	17/4	/	/	21	4.95	16/5	/	/	AR	Accuracy	PLD (11 points)	2	1.07	0.11	0.05	8.5	0.8
Eye-tracking																			
Annaz et al. [26]	17	5.583	/	/	/	17	5.5	/	/	/	PV	% fixations	PLD	6	1.19	0.13	0.06	8.5	0.73
	17	5.583	/	/	/	17	5.5	/	/	/	PV	% fixations	PLD	6	1.75	0.16	0.07		
Burnside et al. [66]	16	5.22	16/0	/	/	16	3.98	8/8	/	/	PV	% fixations	PLD (13 points)	6	0.29	0.12	0.06	8.5	0.82
Fujioka et al. [28]	21	27.6	21/0	99.8	96.4	35	25.2	35/0	/	/	D	% fixations	PLD	20	0.32	0.08	0.04	8	0.8
Fujisawa et al. [29]	15	4.824	12/3	/	/	58	4.008	27/31	/	/	D	% fixations	PLD	20	− 0.2	0.08	0.03	8	0.73
Nackaerts et al. [15]	12	34.9	7/5	111.5	105.7	12	31.5	7/5	115.5	115.3	ER	Fixation time	PLD (12 points)	3	3.27	0.38	0.13	8	0.8
	12	34.9	7/5	111.5	105.7	12	31.5	7/5	115.5	115.3	AR	Fixation time	PLD (12 points)	3	2.58	0.29	0.11		
EEG																			
Bernier et al. [67]	14	23.6	0/14	114	107.6	15	26.7	0/15	108.9	105.4	PV	mu-power ratio (8–13 HZ)	FLD	2.5–20	0.88	0.14	0.07	8.5	0.84
Bernier et al. [68]	19	6.4	18/1	118.3	112	19	6.9	17/2	95.5	96.3	PV	mu-power ration (8–13 HZ)	FLD	6	− 0.06	0.11	0.05	9	0.77
Hirai et al. [69]	12	15.7	11/1	/	/	12	16.5	11/1	/	/	PV	Amplitude N100	PLD	0.990	− 0.21	0.16	0.08	8.5	0.77
	12	15.7	11/1	/	/	12	16.5	11/1	/	/	PV	Amplitude N100	PLD	0.990	0.71	0.17	0.08		
Dumas et al. [70]	10	33.9	7/3	/	/	30	28.7	14/16	/	/	PV	mu-power ratio (8–13 HZ)	FLD	6	3.29	0.26	0.08	8.5	0.73
	10	33.9	7/3	/	/	30	28.7	14/16	/	/	PV	mu-power ratio (8–13 HZ)	FLD	6	2.83	0.23	0.08		
	10	33.9	7/3	/	/	30	28.7	14/16	/	/	PV	mu-power ratio (8–13 HZ)	FLD	6	2.76	0.22	0.07		
	10	33.9	7/3	/	/	30	28.7	14/16	/	/	PV	mu-power ratio (8–13 HZ)	FLD	6	0.05	0.13	0.06		
	10	33.9	7/3	/	/	30	28.7	14/16	/	/	PV	mu-power ratio (8–13 HZ)	FLD	6	1.95	0.18	0.07		
	10	33.9	7/3	/	/	30	28.7	14/16	/	/	PV	mu-power ratio (8–13 HZ)	FLD	6	-0.33	0.13	0.06		
	10	33.9	7/3	/	/	30	28.7	14/16	/	/	PV	mu-power ratio (8–10 HZ)	FLD	6	0.17	0.13	0.06		
	10	33.9	7/3	/	/	30	28.7	14/16	/	/	PV	mu-power ratio (8–10 HZ)	FLD	6	0.79	0.14	0.06		
	10	33.9	7/3	/	/	30	28.7	14/16	/	/	PV	mu-power ratio (8–10 HZ)	FLD	6	− 0.64	0.13	0.06		
	10	33.9	7/3	/	/	30	28.7	14/16	/	/	PV	mu-power ratio (8–10 HZ)	FLD	6	− 0.6	0.13	0.06		
	10	33.9	7/3	/	/	30	28.7	14/16	/	/	PV	mu-power ratio (8–10 HZ)	FLD	6	− 0.9	0.14	0.06		
	10	33.9	7/3	/	/	30	28.7	14/16	/	/	PV	mu-power ratio (8–10 HZ)	FLD	6	− 1.31	0.15	0.06		
	10	33.9	7/3	/	/	30	28.7	14/16	/	/	PV	mu-power ratio (11–13 HZ)	FLD	6	4.96	0.44	0.10		
	10	33.9	7/3	/	/	30	28.7	14/16	/	/	PV	mu-power ratio (11–13 HZ)	FLD	6	4.92	0.43	0.10		
	10	33.9	7/3	/	/	30	28.7	14/16	/	/	PV	mu-power ratio (11–13 HZ)	FLD	6	5.52	0.51	0.11		
	10	33.9	7/3	/	/	30	28.7	14/16	/	/	PV	mu-power ratio (11–13 HZ)	FLD	6	3.98	0.33	0.09		
	10	33.9	7/3	/	/	30	28.7	14/16	/	/	PV	mu-power ratio (11–13 HZ)	FLD	6	− 4.33	0.36	0.09		
	10	33.9	7/3	/	/	30	28.7	14/16	/	/	PV	mu-power ratio (11–13 HZ)	FLD	6	1.9	0.17	0.07		
Raymaekers et al. [38]	20	11.158	18/2	103.2	/	19	10.719	14/5	112.7	/	PV	mu-power ratio (8–13 Hz)	FLD	80	0.47	0.1	0.05	9	0.8
	20	11.158	18/2	103.2	/	19	10.719	14/5	112.7	/	PV	mu-power ratio (8–13 Hz)	FLD	80	0.48	0.1	0.05		
	20	11.158	18/2	103.2	/	19	10.719	14/5	112.7	/	PV	mu-power ratio (8–13 Hz)	FLD	80	0.91	0.11	0.05		

*N* sample size, *FSIQ* full-scale IQ, *NVIQ* non-verbal IQ, *AR* action recognition, *D* BM detection or , *ER* emotion recognition, *PV* passive viewing, *FLD* full-light display, *PLD* point-light display, *d'* sensitivity index, *g* Hedges’ *g*, *var*. *g* estimated variance of *g*, *SE*(*g*) estimated standard error of *g*, *WoE* weight of evidence, *SQA* standard quality assessment score

^▲^ Papers that only reported performance index (accuracy/RT) or in addition to other findings

**Table 2:** Summary of fMRI studies

Author(s) (year)	ASD sample					NT sample					Paradigm	Measure	Stimuli	Duration (s)	Contrast		Areas of activation	WoE	SQA
	N	Age	Sex ratio	FSIQ	NVIQ	N	Age	Sex ratio	FSIQ	NVIQ					Task	Groups			
Alaerts et al. [71]	15	21.7	15/0	107.9	105.6	15	23.3	15/0	114.8	109.1	ER	WB	PLD (12 points)	3s	EM>fix	NT>ASD	L IPL, R MTG-pSTS, L MOG, L MTG-pSTS	8	0.7^▲▲^
Alaerts et al. [30]	15	21.7	15/0	107.9	105.6	15	23.3	15/0	114.8	109.1	ER/D	WB	PLD (12 points)	4s	EM>fix	NT>ASD and ASD>NT	None	8.5	0.79^▲▲^
Bjorndotter et al. [72]	37	boys: 11.45 girls: 10.73	27/10	boys: 91.33 girls: 98.8	/	38	boys: 11.52 girls: 11.51	25/13	boys: 105.46 girls: 93.85	/	PV	WB	PLD (16 points)	24s	BM>SCR	NT>ASD	R FFG, L FFG, L MTG, L IFG, L Cerebellum	8.5	0.8^▲^
Freitag et al. [31]	15	17.5	13/2	101.2	93.3	15	18.6	13/2	112.1	106.8	D	WB	PLD (15 points)	1.5s	BM>SCR	NT>ASD	R Calcarine sulcus, R Parieto-occipital sulcus, R Central sulcus/Postcental gyrus, R Postcentral gyrus/ Postcentral sulcus, R Postcentral sulcus/ IPL, R IPL, R MTG/STS, R Insula, R ACG, R MedFG, R MFG, L Central sulcus/ postcentral gyrus, L IPL, L FFG, L STG, L Claustrum, L ACG	8.5	0.81^▲▲^
Grezes et al. [32]	12	26.6	10/2	102	/	12	21	12/0	119	/	AR/ER	WB	FLD	3s	Dynamic (fear & neutral) vs Static	NT>ASD	R TPJ/STG, R ITG (MT), L ITG, R medSFG, R STG (middle part), R Precentral gyrus, L IFG, R Precaneus, R MFG, R ITG (MT), R FFG/ Cerebellum	9	0.85^▲^
Jack et al. [73]	15	14.2	13/2	110.53	110.33	15	13.8	11/4	112.27	107.6	PV	WB	FLD	7-9s	Hand vs baseline	ASD>NT	R Transverse Temporal Gyrus, L Superior Temporal Gyrus, L Precaneus, R Anterior Cingulate, L Cingulate Gyrus	9	0.9^▲^
Jack et al. [74]	35	14.49	27/8	97.6		62	17.3	26/36	101.61		PV	Cerebellum	PLD (15 points)	24s	BM>SCR	NT>ASD and ASD>NT	None	8	0.78^▲^
Kaiser et al. [75]	25	11.8	20/5	100.2	98.2	NT: 17 US: 20	NT: 10.9 US: 11.3	NT: 12/5 US: 9:11	NT: 114.1 US: 115.8	NT: 110.1 US: 113. 8	PV	WB	PLD (16 points)	~24s	BM>SCR	NT>ASD	L vlPC, vmPC, R PTS, R Amygdala, R FFG, L FFG	8.5	0.75^▲^
Koldewyn et al. [57]	16	15.4	14/2	110.6	106.7	16	15.6	14/2	118.6	112.6	D	WB	PLD (13 points)	2s	BM>COH	NT>ASD	R Insula,, Bilateral Caudate, Bilateral Pulvinar; R Intraparietal sulcus, R AG, R STS; R IFS, R MFG, R IFG; L Intraparietal Sulcus, L AG, L STS; Anterior Cingulate Sulcus and Gyrus.	8.5	0.84^▲▲^
																ASD>NT	R ITG/IOG (2 clusters)		
Marsh et al. [76]	18	33	/	110.22	104.4	19	32.2	/	113.89	113.4	PV	WB	FLD	24s	Hands>shapes	NT>ASD	L Middle Cingulate extending to Supplementary Motor Area, L Fusiform/Lingual Gyrus	8	0.73^▲^
Yang et al. [77]	31	10.86	31/0	98.1	96.65	17	10.92	17/0	104.1	103.7	PV	WB	PLD (16 points)	~24s	BM>SCR	NT>ASD	R AG, R FFG, R Hippocampus, R IOG, R MOG, R IPG, R ITG, R MTG	8	0.9^▲^

*N* sample size, *FSIQ* full-scale IQ, *NVIQ* non-verbal IQ, *AR* action recognition, *D* BM detection, *ER* emotion recognition, *PV* passive viewing, *WB* whole brain analysis, *FLD* full-light display, *SCR* scrambled BM, *COH* coherent dot motion, *PLD* point-light display, *WoE* weight of evidence, *SQA* standard quality assessment score, *L* left, *R* right, *IPL* inferior parietal lobule, *AG* angular gyrus, *FFG* fusiform gyrus, *IOG* inferior occipital gyrus, *MOG* middle occipital gyrus, *ITG* inferior temporal gyrus, *MTG* middle temporal gyrus, (p)*STS* (posterior) superior temporal sulcus, (med)SFG (medial)superior frontal gyrus, *IFG* inferior frontal gyrus, *MFG* middle frontal gyrus, *vlPC* ventrolateral prefrontal cortex, *PTS* posterior temporal sulcus, *vmPC* ventromedial prefrontal cortex, *TPJ* temporo-parietal junction, *STG* superior temporal gyrus, *ACG* anterior cingulate gyrus, ^*▲▲*^ score represents the total score obtained from the behavioural quality assessment plus a score given for the fRMI protocol, ^*▲*^ score represents the relevant questions from the quality assessment measure + a score for the fMRI protocol

Data on performance in the sense of descriptive statistics, *t* values or effect sizes (*d*), were extracted from each paper. Effect sizes for thresholds, accuracy, sensitivity indices, error rates and reaction times were recorded from the behavioural studies. The areas of activation with contrasts of ASD > NT or NT > ASD were recorded from the fMRI studies and fixations or proportion of fixations were collected from the eye-tracking experiments. Eye-tracking studies included preferential looking paradigms in which percentage fixations were recorded as an indication of preference for one display, i.e. BM, over another, i.e. inverted BM. Differences in EEG-recorded activation between the NT and ASD groups were extracted from the EEG experiments, along with the specific frequencies and electrodes used. Additionally, the following variables were extracted to allow for a complete account of the included studies and quality assessment:

1. Diagnosis confirmation criteria
2. Type and number per diagnosis category (where available)
3. Additional diagnoses reported
4. Verbal IQ and other cognitive abilities that were not measured by a complete IQ assessment
5. Length of presented stimulus

### Quality assessment

Risk of bias for behavioural, eye-tracking and EEG studies was assessed by two independent reviewers using the standard quality assessment (SQA) criteria for evaluating primary research papers from various fields for quantitative studies [78]. The checklist contains 14 items. Items 5 (If interventional and random allocation was possible, was it described?), 6 (If interventional and blinding of investigators was possible, was it reported?), 7 (If interventional and blinding of subjects was possible, was it reported?) were not used as they refer to the use of interventions which are not applicable for the studies reviewed here. Each of the remaining 11 items can receive 2 points if the assessed study fulfils the criteria; 1 point if it partially fulfils the criteria and 0 points if it does not fulfil the criteria at all. A summary score was calculated for each paper by adding the total score and dividing it by the total possible score. The total score after excluding the previously mentioned three items is calculated with Eq. 1. One study [56] provided only descriptive information of results (no inferential statistics) and was judged on fewer items (Q1–4, Q8–9, Q13–14).

1 $$ 28-\left(3\left[\mathrm{excluded}\ \mathrm{items}\right]\ast 2\right)=22 $$

Eight studies were chosen at random to pilot the quality assessment. Disagreements were discussed and all papers were re-evaluated. An initial comparison was then done between the reviewers’ scores. It was found that most disagreements were on item 12 (‘Controlled for confounding?’). This item was discussed, and the papers were re-evaluated for that item. Disagreements of more than 3 points difference were further discussed on an item-by-item basis. Final comparison of all papers resulted in 18 papers upon which the reviewers completely agreed on the total score. There was no more than a two-point absolute difference between the reviewers’ scores for the remaining papers. Thus, the scores for these papers were averaged across both reviewers. Differences between the two reviewers were mostly in the assignment of full or partial points for the items, which was also evident in the original piloting of the scales during its development [78]. Overall, the disagreement between the reviewers in the quality score given to each study was quite low with small variability—0.038 (SD = 0.035, min-max [0–0.091]). In total, 47 papers were evaluated. The overall SQA score given to all papers was medium/high—0.792 (SD = 0.065, min-max [0.636–0.955]).

We were unable to locate a standardised quality assessment measure that would allow us to assess the quality of fMRI papers. Thus, the assessment was done using relevant criteria from the SQA. Specifically, questions related to the analysis and results were excluded and the fMRI methodology was assessed for robustness. This was done collaboratively by the authors.

For the fMRI studies, which included an analysis of behavioural performance, the fMRI part of the analysis was disregarded initially, and the rest was assessed using the standard SQA procedure described above. This was done to provide a comparable score across the studies that incorporated behavioural performance and to allow for the inclusion of the quality measures as a predictor variable in the analysis. Afterwards, their fMRI protocols and analyses procedures were assessed for methodological robustness by the third and first author. The originally agreed upon score from the SQA was added to the score given for the methodological robustness and a new average quality score was calculated. For the fMRI papers that did not contain a behavioural paradigm, we used the relevant questions from the SQA (Q1–Q4, Q9 and Q12–Q14). Additionally, their protocols and analyses procedure were assessed for robustness. These scores were added and a composite score was given. Thus, it is important to underline that the quality scores for the fMRI papers are not directly comparable with the rest of the papers. The quality assessment scores for each study are presented in Tables 1 and 2.

Additionally, in order to evaluate the quality of the evidence included, we have further conducted a weight of evidence analyses [79]. The majority of shortcomings that were identified came from a non-randomised procedure or not including all sample characteristics. Details of this analysis are shown in Additional file 2. It indicates that despite their shortcomings, the included studies provide good quality and relevant evidence in support of our conclusions.

### Statistical analysis

The following analysis procedure was applied to the behavioural, eye-tracking and EEG experiments. For each included paper, the descriptive statistics, *t* values or Cohen’s *d* were used to calculate Hedges’ *g* as the common representation of effect size for all studies. All the calculations and transformations were done by firstly calculating Cohen’s *d* and its variance. A correction for small sample size was applied to get the unbiased estimate of Hedges’ *g*. The variance of *g* was estimated based on the sample sizes of each study. All the calculations were done using the R package *compute.es* [80] in R(v3.4.1) [81] and RStudio (v.1.1.453) [82]. A precision index was calculated for each study as the inverse of the variance (1/variance). Positive Hedges’ *g* corresponded to higher scores (better performance) in NT, when compared to ASD. Five top outlier outcomes were identified using a boxplot. An analysis of the initial model with and without the outliers showed that without the outliers, the variance between the studies reduced by a factor of 1.3 and the residual estimates reduced by a factor of five. Thus, all statistical analyses within this paper report the results without the outliers.

Six studies provided RT data. Since a previous meta-analysis [21] showed that RT outcomes tap into different processes in comparison to the rest of the extracted outcomes, they were analysed separately from the rest of the behavioural outcomes. Two top and one bottom outlier were identified using a boxplot. As above, the variance between the studies reduced without the outliers, and the residual estimate reduced by a factor of 3.6. Thus, all statistical analyses report the results without the outliers.

Since papers rarely report only one outcome and/or have only one experiment from which an effect size can be extracted, the traditional (two-level) meta-analysis is not appropriate due to the dependencies that come from using the same subjects or having the same researchers conduct the study [83–85]. Therefore, the analysis was extended to a three-level meta-analysis, which takes into account the variance due to the variation of the effect sizes included; the variance that occurs within the same study and the variance that occurs between the studies [84]. Therefore, the three-level analysis estimates these three variance elements. The error only linear model with no moderators as given by Cheung [83] is shown in Eq. 2:

2 $$ {g}_{jk}={\alpha}_0+{u}_k+{u}_{jk}+{e}_{jk} $$

Where *g*_jk_ is the effect size for outcome *j* from study *k* and is represented by Hedges’ *g*; *α*_0_ is the grand mean of all effect sizes across studies; *u*_*k*_ represents the deviation of the average effect in study *k* from the grand mean; *u*_*jk*_ is the deviation of effect *j* in study k from the average effect of study *k*; and finally *e*_*jk*_ is the residual variation not explained by the previously defined variances [83]. This random effects model is then extended by including moderators. A series of meta-analyses were conducted to investigate the effect of one or a combination of more than one of the following covariates: age, sex ratio, full-scale intelligence quotient (FSIQ) and non-verbal intelligence quotient (NVIQ) for each group, as well as the paradigm and the stimuli. When moderators are added to the analysis, there are two sets of effect sizes that need to be kept in mind. The first set of effect sizes are the difference between ASD and NT at that level of the moderator (or combination of moderators). These are presented in Tables 4 and 5. The second set of effect sizes are the ones which represent the size of the difference between the different levels. For example, a positive effect size will indicate that at the first level of the moderator, the difference between ASD and NT is larger than at the second level. Negative effect sizes here represent that there is a larger effect at the second/third/etc. level than at the previous level.

The parameter estimation was done using maximum likelihood, implemented in the mixed procedure in the statistical package SAS (release 9.04.01, [86]). Due to the imbalance of studies when the predictor variables were added, the Satterthwaite method was used to calculate the denominator degrees of freedom [87]. Additionally, to investigate the effects at each level of the categorical variables, a least square means procedure was applied.

To assess heterogeneity, the *I*^*2*^ statistic [88] was calculated. Since we are using a three-level analysis and potential heterogeneity can occur at the second or the third level, we used the modified formulas provided by Cheung [83]. The *I*^2^ statistic was calculated only for the initial model, the model with the paradigm as a moderator and the model that included both paradigm and age as moderators. This was done because these three models contained the same studies and thus the effect of the moderators on the heterogeneity could be compared. The calculations for level 2 ${I}_{(2)}^2$ and level 3 ${I}_{(3)}^2$are shown in Eq. 3 below. ${I}_{(2)}^2$ and ${I}_{(3)}^2$represent the proportion of variation which can be attributed to the between and within studies respectively.

3 $$ {I}_{(2)}^2=\frac{{\hat{u}}_{(2)}^2}{{\hat{u}}_{(2)}^2+{\hat{u}}_{(3)}^2+\overset{\sim }{v\ }} $$

4 $$ {I}_{(3)}^2=\frac{{\hat{u}}_{(3)}^2}{{\hat{u}}_{(2)}^2+{\hat{u}}_{(3)}^2+\overset{\sim }{v\ }} $$

Where ${\hat{u}}_{(2)}^2$is the between study variance calculated from the model, ${\hat{u}}_{(3)}^2$ is the within study variance calculated by the model and $\overset{\sim }{\nu }$ is the typical within study variance calculated by Eq. 4 as suggested by Higgins ant Thompson [88].

5 $$ \overset{\sim }{v}=\frac{\sum {w}_i\left(k-1\right)}{{\left(\sum {w}_i\right)}^2-\sum {w_i}^2} $$

Where *w* is the inverse variance and *k* is the number of studies.

Publication bias was assessed with Egger Regression [89] and the Trim and Fill method [90] using a two-level random effects model. The analysis was performed using a SAS macro created by Rendina-Gobioff and Kromrey [91].

### ALE analysis of fMRI studies

To analyse the fMRI data, activation likelihood estimation (ALE) in GingerALE v3.0.2 [92–94] was employed. Foci from the between group contrasts, which had reached statistical significance, were first extracted from the studies and converted where necessary into Talairach space using GingerALE. When both whole-brain and region-of-interest analyses were performed, and coordinates were available, the ones from the whole-brain analysis were used. In ALE, the activation foci are shown as a three-dimensional Gaussian probability density function, centred at the specified coordinates. The spatial overlap of these distributions across the different studies and the spatial uncertainty due to inter-subject and inter-experiment variability are then computed. This results in activation maps, which can be seen as summaries of the results of a specified study after considering the spatial uncertainty present. Through the combination of these maps, the convergence of activation patterns across studies can be calculated. This is confined to a grey matter shell and above chance clustering between the studies is calculated as a random-effects factor [93]. We performed ALE analysis for the NT > ASD contrast only, since only two studies found differences at the ASD > NT contrast [57, 73]. Only two studies [32, 71] provided data for emotion detection/identification paradigms, thus this was not analysed separately. Although, our initial intent was to investigate the effects of age, the small amount of studies that provided information about the differences between the ASD and the NT group would not allow for a separate investigation, without introducing spurious results and further complicating the mixed literature in the field. Thus, the readers should keep in mind that the ALE analysis and the output produced contains research from both children/adolescents and adults as well as emotion and BM detection/observation paradigms. Using the recommended thresholding procedure—cluster defining threshold of 0.001 and cluster-wise family-wise error correction of 0.05, we were not able to identify any significant clusters. An exploratory analysis is reported where we used an uncorrected *p* value of 0.001 and maximum cluster size of 200 mm^3^.

Data used for the analysis is deposited in a data repository, the link and reference to which will be added post acceptance, to allow for masked review.

## Results

The initial (November 2017) study search returned 793 records. The output from all databases was combined and duplicates were removed using two strategies. Initially, R software was used to remove duplicate records that appeared in the same format between the searches. Then, the articles were screened by hand to remove additional duplicates. This resulted in a total of 516 records. At the second search (May 2019), 124 records were identified and Rayyan software was used [95]. Out of those 45 were identified as duplicates from the previous search and 18 were identified as duplicates between the databases. This resulted in a total of 61 records.

**Fig. 1:**
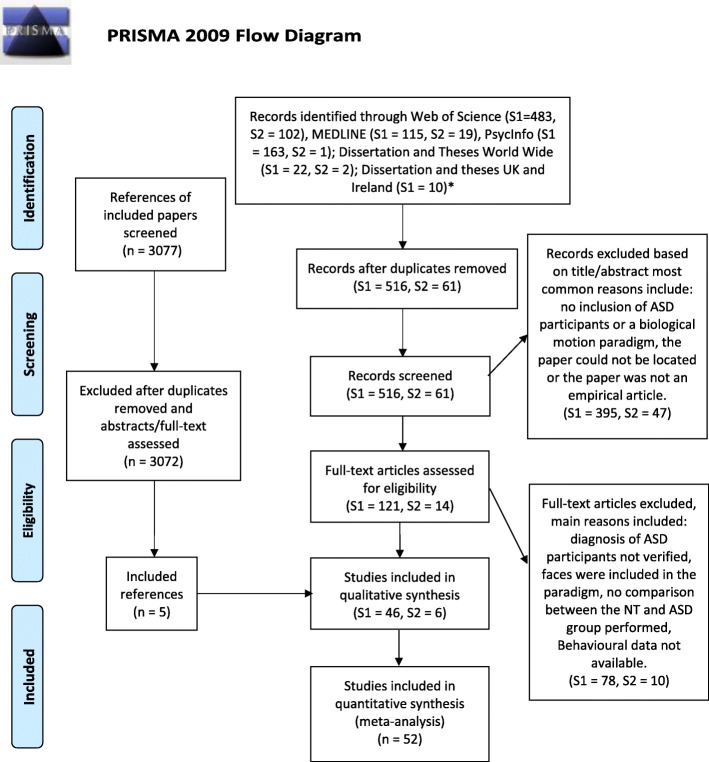
PRISMA flow diagram representing the selection/inclusion/exclusion process*.* Adapted from Moher et al. [96]. * Note that the second search did not look into Dissertation and Theses UK & Ireland, as it was covered by Dissertation and Theses Worldwide in the previous search

The selection process resulted in a set of 47 papers. Five further records were identified from the references of the included papers. From these 35 contributed to the behavioural studies category, five to the eye-tracking category, five to the EEG category and 11 to the fMRI category. An overview of the inclusion/exclusion process is shown in the PRISMA flow diagram in Fig. 1 below.

The included studies and their descriptive information can be seen in Table 1 (behavioural, eye-tracking and EEG) and Table 2 (fMRI). The two tables also show the effect sizes for each study, their variance and standard error, their weight of evidence score and their quality assessment score.

This meta-analysis examined 52 papers, which contributed 80 (11 RT) behavioural effect sizes, seven eye-tracking effect sizes, 25 EEG effect sizes and 76 fMRI Foci. The sample size for the behavioural sample included 1742 subjects (ASD: 867, NT: 875). The complete eye-tracking sample included a total sample of 217 participants (ASD: 65, NT: 122). The EEG sample had a total sample of 170 participants (ASD: 75, NT: 95). The fMRI sample had a total sample of 483 participants (ASD: 234, NT: 249). Participant characteristics from all studies (including studies considered outliers in the analyses) are shown in Table 3.

**Table 3:** Participant characteristics in each type of analysis

Paradigm (number of studies)	Included studies	ASD					NT					N
		Age (SD)	Proportion of females mean (SD)	FSIQ mean (SD)	NVIQ mean (SD)	N	Age (SD)	Proportion of females mean (SD)	FSIQ mean (SD)	NVIQ mean (SD)	N	
Behavioural (*N* = 35)	[9–17, 19, 20, 22, 23, 30, 31, 45, 46, 48–65]	19.86 (10.75)	19.15 (27.69)	106.3 (9.76)	98.28 (13.58)	867	19.46 (10.28)	23.38 (23.85)	111.93 (7.42)	105.28 (15.03)	875	1742
RT (*N* = 6)	[10, 22, 31, 50, 59, 62]	16.71 (5.76)	9.51 (8.43)	105.63 (3.84)	96.25 (11.66)	123	17.33 (5.76)	10.3 (8.19)	116.07 (4.73)	108.56 (3.36)	135	258
Eye-tracking (*N* = 5)	[15, 26, 28, 29, 66]	15.63 (14.5)	15.42 (19.89)	105.65 (8.27)	101.05 (6.58)	81	14.04 (13.28)	36.28 (24.69)	115.5 (0)	115.3(0)	138	217
EEG (*N* = 5)	[38, 67–70]	18.15 (10.85)	28.61 (38.57)	111.83 (7.78)	109.8 (3.11)	75	17.90 (9.6)	35.49 (36.45)	105.71 (9.05)	100.85 (6.43)	95	170
fMRI (*N* = 11)	[30–32, 57, 71–77]	18.03 (7.05)	12.91 (9.99)	103.76 (5.8)	102.6 (5.84)	234	17.54 (6.60)	17.76 (21.99)	111.73 (6.41)	109.57 (3.34)	249	483

### Behavioural performance

#### **O**verall

**Fig. 2:**
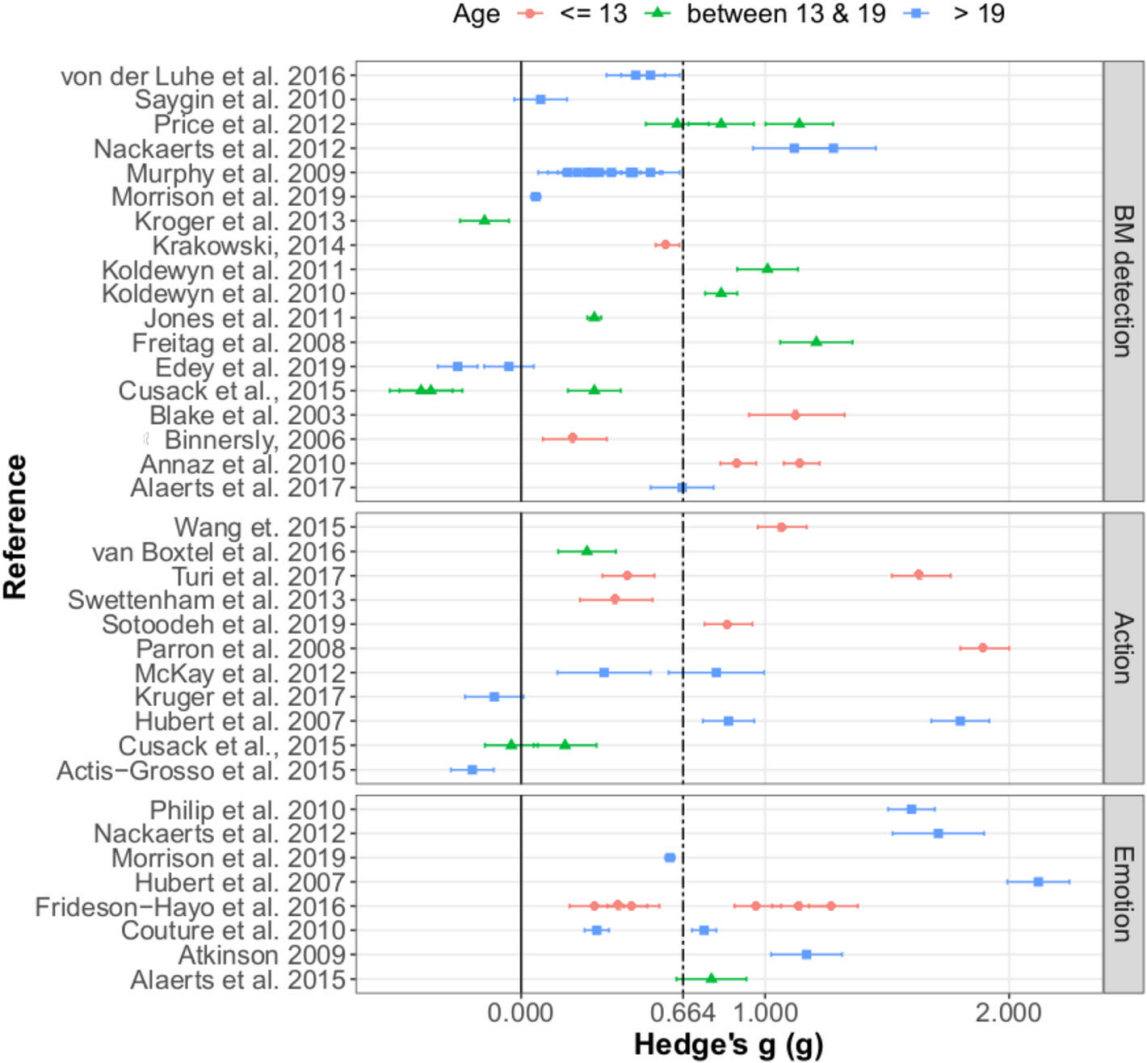
Forest plot showing the effect sizes (Hedge’s g) from each study and its standard error as the error bars of the points. Different colours/shapes represent the different age categories (red/circle—bellow or equal to 13; green/triangle—between 13 and 19; blue/square—older than 19) and the graph is split by paradigm. Solid line represents no effect; positive effect sizes represent instances where ASD participants performed worse than NT; dot-dashed line represents the effect size extracted from the initial model (*g* = 0.6639)

The random effects three-level analysis of the overall sample revealed a mean estimated effect size *g* = 0.6639 [SE = 0.0923, 95% CIs 0.4759–0.8520] *t*(31.6) =7.2, *p* < 0.0001, which represents a medium effect [97]. Overall, this suggests that ASD participants were less accurate, less sensitive or produced more errors when asked to detect or interpret biological motion in comparison to NT individuals. The between study variance (*u*_*k*_ = 0.1965 [SE = 0.072], *Z* = 2.73, *p* = 0.0032) and the within study variance (*u*_*jk*_ = 0.0701 [SE = 0.07], *Z* = 1, *p* = 0.1584) show that variance occurred mostly between the studies. The heterogeneity at level 2 is ${I}_{(2)}^2$ = 0.424, which argues for low to moderate heterogeneity and at the third level ${I}_{(3)}^2$ = 0.0539, which falls under the category of low heterogeneity. The variance component was significant only between studies, indicating that the results varied more between than within studies, which mirrors the heterogeneity measures. It can be seen in Fig. 2 that the effect sizes of the studies and their confidence intervals cluster around the estimated effect size from the model, and only a few studies cross the line of no difference. Studies included in this analysis are as follows: [9–14, 16, 17, 19, 20, 22, 23, 30, 31, 45, 46, 48–51, 53–55, 57–65, 98].

#### Quality

An exploratory meta-analysis was run with the quality given to the studies using the quality assessment tool. However, there did not appear to be an effect of the quality of the studies on the results—*F*(1,25.6) = 1.79, *p* = 0.1932. It has to be pointed out that most studies received quite high scores on the quality assessment measure, which could potentially explain the absence of an effect. However, the inclusion of quality did reduce the variation between the studies (*u*_*k*_ = 0.1754 [SE = 0.0696], *Z* = 2.52, *p* = 0.0058), despite slightly increasing the within-studies variance (*u*_*jk*_ = 0.0753 [SE = 0.0767], *Z* = 0.98, *p* = 0.1631). For this reason, quality scores were added as a covariate within the rest of the analyses [99]. For most cases, its inclusion either decreased covariance between the studies or had no qualitative effect. All studies from the overall analysis were included in this analysis.

#### Stimuli

To see whether the type of stimuli—full-light or visually sparse (e.g. PLDs)—had an effect on participant’s performance, the stimuli type was added as a moderator variable. One paper included both full-light displays, and point light displays and thus was excluded [19]. This reduced the number of effect sizes for this meta-analysis only from 64 to 63. The analysis showed that there was no overall effect of the type of stimulus used—*F*(1,24.9) = 0.91, *p* = 0.3493. Additionally, the effects for full-light displays and PLDs were both significantly above 0—*g* = 0.9055 [SE = 0.3055, 95% CIs 0.2759–1.5351] *t*(24.7) = 2.96, *p* = 0.0066 and *g* = 0.5842 [SE = 0.1006, 95% CIs 0.3778–0.7905] *t*(27) = 5.81, *p* < 0.0001, respectively. Full-light displays showed larger variance, potentially due to a smaller number of studies (*N* = 10).

#### Paradigm

There was an overall effect of the type of paradigm used—*F*(2,61.5) = 8.70, *p* = 0.0005. There was a significant effect of each paradigm type as shown in Table 4, indicating that participants with ASD performed worse than the NT in all paradigms. More interesting are the pairwise differences in performance between the paradigms. The difference in performance between the detection of coherent BM and action recognition/categorisation was not significant (*g* = − 0.0222 [SE = 0.1646, 95% CI − 0.3511, 0.3067], *t*(63.8) = − 0.13, *p* = 0.8933). However, there were significant differences between the detection of BM and emotion recognition/categorisation (*g* = − 0.5647 [SE = 0.1373, 95% CIs − 0.8399, − 0.2896], *t*(55.8) = − 4.11, *p* = 0.0001), as well as between action recognition/categorisation and emotion recognition/categorisation (*g* = − 0.5426 [SE = 0.1922, 95% CIs − 0.9268, − 0.1583], *t*(62.4) = − 2.82, *p* = 0.0064). In both situations, ASD participants showed decreased performance in comparison to NT participants in the emotion recognition/categorisation paradigms than in any of the other two. After the paradigm was added as a moderator, the variance reduced slightly at the between studies level (*u*_*k*_ = 0.1537) and disappeared at the within study level (*u*_*jk*_ = 0). Similarly, the heterogeneity decreased from the initial model for level 2 and for level 3 (${I}_{(2)}^2$ = 0.3319 and ${I}_{(3)}^2$ = 0). Finally, quality scores did not show a significant effect at this stage *F*(1,29) = 3.48, *p* = 0.0724. All studies from the overall analysis were included in this analysis.

**Table 4:** Simple effects for each paradigm

Paradigm	ES	*g*	SE	Lower CI	Upper CI	*df*	*t*	*p>t*
1	36	0.5041	0.1012	0.2989	0.7093	36.4	4.98	< 0.0001*
2	17	0.5274	0.1476	0.2316	0.8233	54.7	3.57	0.0007*
3	14	1.0618	0.1422	0.7773	1.3462	60.1	7.47	< .0001*

1—Detection of BM in noise and recognition in comparison to other stimuli; 2—action recognition/categorisation; 3—emotion recognition/categorisation. *ES* number of effect sizes, *g* Hedges’ *g*, *SE* standard error, *df* degrees of freedom

* Significant at 0.05

#### Paradigm and age

Next, both age and paradigm were included in the analyses and were allowed to interact. A meta-analysis with paradigm and age showed no main effects of paradigm (*F*(2, 44.2) = 2.10, *p* = 0.1348) and no interaction between age and paradigm (*F*(2, 34.3) = 1.44, *p* = 0.2426). However, there was a significant main effect of age (*F*(2,29) = 3.35, *p* = 0.0492). Simple effects of each age group are reported in Table 5. Visual representation of the effect sizes is shown in Fig. 2, where the graph is separated by paradigm and the different age groups are colour/shape coded. Note that only one effect was recorded for adolescents in the emotion category.

**Table 5:** Simple effects for each age group

Age	ES	*g*	SE	Lower CI	Upper CI	*df*	*t*	*p>t*
1	17	0.9528	01463	0.6443	1.2614	17	6.51	< 0.0001*
2	15	0.4215	0.1963	0.02701	0.8160	49	2.15	0.0368*
3	35	0.5000	0.1089	0.2751	0.7249	23.6	4.59	0.0001*

Age: 1—≤ 13, 2—> 13 and ≤ 19, 3—> 19. *ES* number of effect sizes, *g* Hedges’ *g*, *SE* standard error, *df* degrees of freedom

* Significant at 0.05

There were no significant differences in the effect size of the ASD-NT difference between adolescents and adults (*g* = − 0.07848 [SE = 0.2178, 95% CIs − 0.5125, 0.7517], *t*(42.4) = − 0.36, *p* = 0.7204). However, there were significant differences in the effect size of the ASD-NT difference between children and adolescents (*g* = 0.5313 [SE = 0.2523, 95% CIs 0.01878, 1.0438], *t*(34.3) = 2.11, *p* = 0.0426) and between children and adults (*g* = 0.4528 [SE = 0.1881, 95% CIs 0.05998, 0.8457], *t*(19.7) = 2.41, *p* = 0.0260). The effects show that in both cases if the tested participants were children, the effects sizes were larger.

After both age and the paradigm were added as moderators the variance between studies reduced even more, with again no variance being attributed to the third level (*u*_*k*_ = 0.0866 and *u*_*jk*_ = 0). Furthermore, the heterogeneity was almost completely accounted for by the moderators (${I}_{(2)}^2$ = 0.1363 and ${I}_{(3)}^2$ = 0).

Additionally, the quality scores showed a significant—*F*(1,30.2) = 8.17, *p* = 0.0076, showing that with the increase of the quality of the study, the smaller the effects were. All studies from the overall analysis were included in this analysis.

#### Sex

The proportion of females in the samples of both ASD and NT participants was included as moderator variables in two smaller meta-analyses. Since several studies did not report information about sex, only 56 effect sizes from 27 studies were included in these analyses. The proportion of females in the ASD sample had no effect on the results (*F*(1, 33.2) = 0.11, *p* = 0.7454) nor did the proportion of females in the NT sample (*F*(1, 29.7) = 0.61, *p* = 0.4402). Studies included in this analysis are as follows: [9–12, 17, 19, 20, 22, 23, 30, 45, 46, 48–50, 53–55, 57–62, 64, 65, 98].

#### Full-scale IQ

Similar to sex, there were several studies that did not report FSIQ for one or both of the groups. For the ones that did report the FSIQ of both ASD and NT participants, FSIQ was also included as a moderator variable in two smaller meta-analyses. These included 18 studies and 30 effect sizes. There was no effect of FSIQ within the ASD sample (*F*(1, 15.9) = 0.02, *p* = 0.8889) nor was there an effect of FSIQ within the NT sample (*F*(1, 30) = 3.98, *p* = 0.0553). Studies included in this analysis are as follows: [11, 14, 17, 19, 20, 22, 30, 31, 48, 53–55, 57, 58, 61, 64, 65, 98].

#### Non-verbal IQ

Only 14 studies and 18 effect sizes included the NVIQ for both the ASD and the NT group. Two smaller meta-analyses were performed using the NVIQ of each group as moderator variables; however, there were no significant effects neither for the ASD NVIQ (*F*(1,12.1) = 0.15, *p* = 0.7012) nor for the NT NVIQ (*F*(1,11.3) = 0.00, *p* = 0.9921). Studies included in this analysis are as follows: [11, 17, 19, 20, 22, 30, 31, 48, 50, 57, 58, 62, 98, 100].

### Publication Bias

**Fig. 3:**
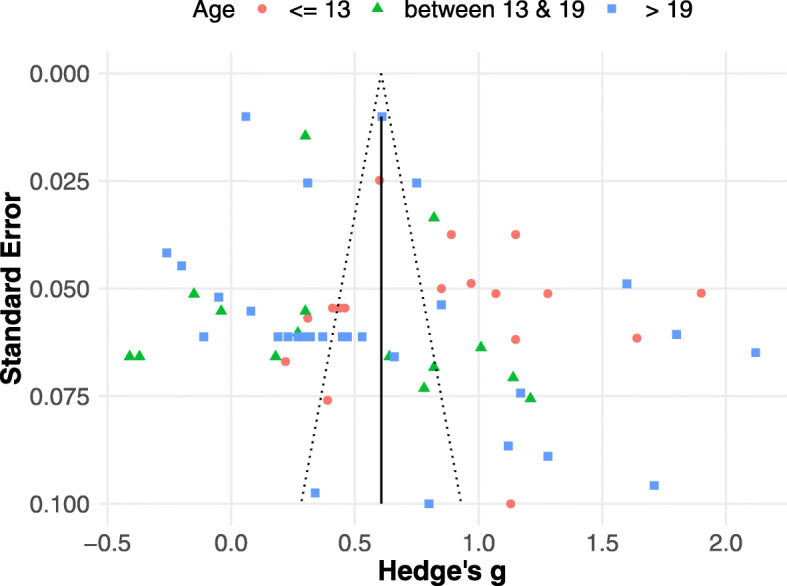
Funnel plot for the behavioural studies. Displays the effect size—Hedge’s g, plotted against the standard error. The vertical line represents the effect size from the overall analysis

To evaluate the possibility of a publication bias, we plotted the behavioural effect sizes against their standard error with a funnel plot (see Fig. 3) [89, 101]. As can be seen by their distribution, there is a wide variety of effect sizes with similar standard errors. Specifically, there appears to be a lack of effect sizes with high standard errors and low effect sizes and low standard errors with high effect sizes, which stems from the relatively small to moderate sample sizes in the studies. The inverted funnel shape, which extends 1.96 standard errors around the overall estimate, should include 95% of the studies. However, one of the assumptions for that interpretation is that the true effect is the same in each study [102]. It is evident from Fig. 3 that 95% of the studies do not fall within the funnel shape. However, we do not make the assumption that the treatment effect is the same in each study. Moreover, we show that the effects vary with age and paradigm. Finally, it is possible that additional variability is added due to the heterogeneous nature of the ASD population.

Besides visual inspection of the funnel plot, the Egger regression method [89] was used to assess the possibility of bias using a random effects model. Egger’s regression detected a risk of publication bias—*t* = 2.5806, *p* = 0.0122. Specifically, there is slight asymmetry in the lower end of the funnel plot, where larger standard errors produced larger effect sizes. For this reason, the Trim and Fill method from Duval and Tweedie [90] was used. Using a standard random effects model, the analysis indicates publication bias in the right tail of the funnel plot, indicating that more studies were published with large effect sizes and large standard errors. This was mirrored by the direction of the effect found in the meta-analysis including the quality assessment scores.

### Reaction time

**Fig. 4:**
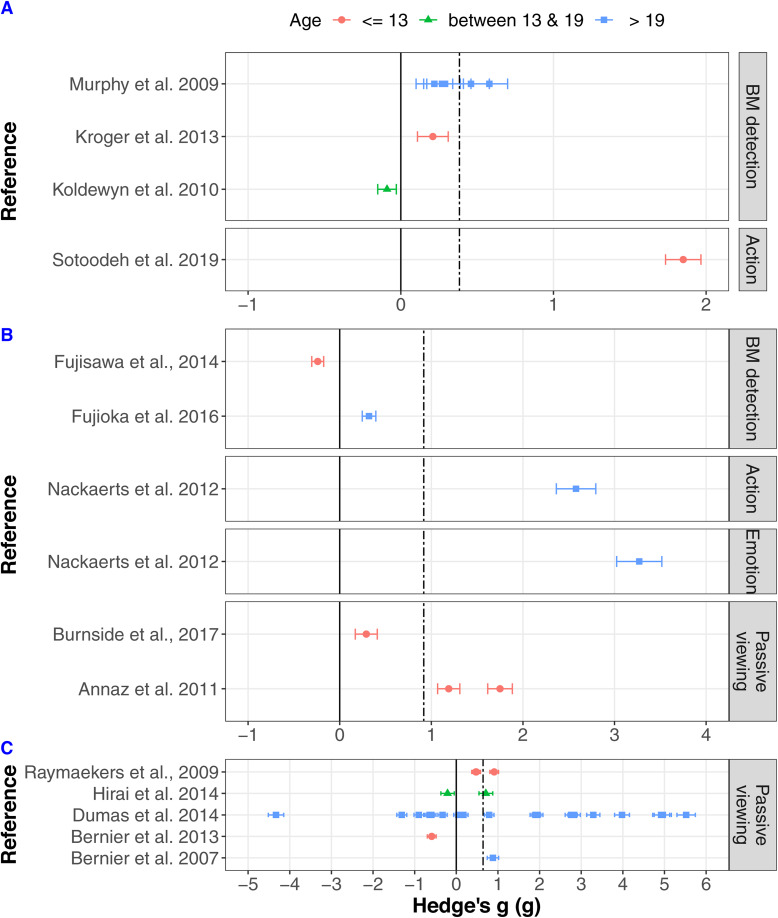
Forest plots showing the effect sizes (Hedge’s g) from each study and its standard error as the error bars of the points. Different colours/shapes represent the different age categories (red/circle—bellow or equal to 13; green/triangle—between 13 and 19; blue/square—older than 19) and the graph is split by paradigm. Solid line represents no effect; positive effect sizes represent instances where ASD participants performed worse than NT; dot-dashed line represents the effect sizes extracted from the initial model. **a** Reaction time data (*g* = 0.384), **b** eye-tracking data (*g* = 0.917) and **c** EEG data (*g* = 0.642)

The random effects three-level analysis of the overall RT sample revealed a mean estimated effect size *g* = 0.384 [SE = 0.1828, 95% CIs − 0.037–0.8055] *t*(8) = 2.1, *p* = 0.0689, which represents a small effect [97]. Overall, this suggests that ASD participants showed non-significantly slower RT in the BM paradigms in comparison to NT individuals. There was no between study variance (*u*_*k*_ = 0) or within study variance (*u*_*jk*_= 0), thus heterogeneity was not calculated. With the removal of outliers, there were only eight effect sizes left, and further moderation analyses were not run [103]. Figure 4a shows the distribution of effect sizes for the reaction time paradigms. Studies included in this analysis are as follows: [10, 22, 59, 62].

### Eye-tracking

As there were only five papers that provided enough information to extract data about effect sizes in eye-tracking experiments, a meta-regression with moderators was not conducted. The five studies contributed a total of seven effect sizes. The overall analysis revealed a mean estimated effect size *g* = 0.9172 [SE = 0.4865, 95% CIs − 0.3552, 2.1896], *t*(4.73) = 1.89, *p* = 0.1214, which represents a large effect, but non-significant [97]. Overall, this means that ASD participants showed less preference for biological motion in comparison to NT individuals; however, it should be noted that it was not significant, which is predicated by the broad confidence intervals around the estimate. The between study variance (*u*_*k*_ = 1.0862 [SE = 0.7841], *Z* = 1.39, *p* = 0.083) and the within study variance (*u*_*jk*_ = 0.0) showed that variance occurred mainly between studies, which was expected due to the small number of studies. However, none were significant indicating consistency between the studies’ results and the results within studies. It is important to point out that due to the small number of studies and the large confidence intervals, these results should be taken with caution. Figure 4b shows the distribution of effect sizes for the eye-tracking paradigms. All studies reported in Table 1 under the eye-tracking subheading are included.

### EEG

There were 25 effect sizes provided by five studies. The overall effect size revealed by the analysis was not significant—*g* = 0.6489 [SE = 0.3271, 95% CIs − 0.02476, 1.3226], *t*(25) = 1.98, *p* = 0.0584. Similar to the eye-tracking results, this showed a medium effect size but due to the small sample size, and the fact that one study contributed 17 of the effect sizes, it is expected that the large confidence intervals would overlap with 0. There was no between or within study variance—*u*_*k*_ *= u*_*jk*_ *=* 0. Figure 4c shows the distribution of effect sizes for the EEG paradigms. Due to the variability that is seen in the frequency that is used, an exploratory analysis, which looks at frequency as a contributing factor to the EEG findings, is reported in Additional file 3. All studies reported in Table 1 under the EEG subheading are included.

### fMRI

**Fig. 5:**
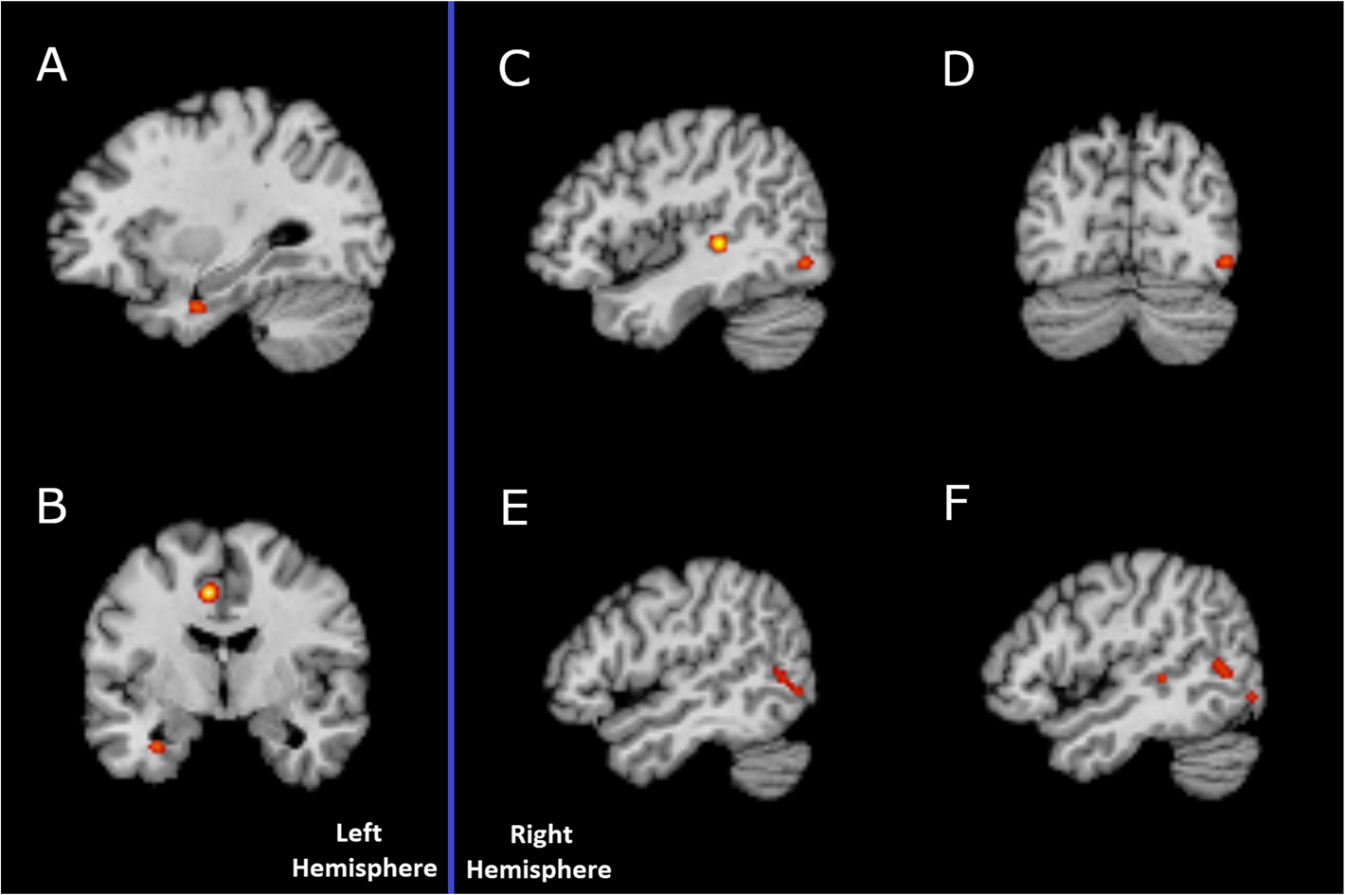
Brain area activation from ALE analysis. **a** Uncus. **b** Central gyrus. **c** Superior temporal gyrus. **d** Middle occipital gyrus. **e** Inferior temporal gyrus. **f** Middle temporal gyrus

The 11 studies that investigated the difference between ASD and NT participants covered emotion recognition and distinguishing between coherent BM PLD and scrambled PLD/fixation baseline or coherently moving dots. Due to the small sample of studies and the fact that two studies did not find any significant brain areas, and one study only found difference in the ASD > NT contrast, all studies were analysed together for the NT > ASD contrast. Only Koldewyn et al. [57] and Jack et al. [73] found differences where ASD participants showed significantly higher activated regions when compared to NT. Since these were the only two studies to show this contrast, no further analysis was done for the ASD > NT contrast. This led to the inclusion of eight studies (62 foci). Due to the small number of included studies, we used the uncorrected *p* values at a level of 0.001 and a minimum cluster size of 200 mm^3^. Table 6 and Fig. 5 present the results from the NT > ASD comparison. Five clusters were identified where the NT participants showed greater activation than the ASD participants. In the left hemisphere, one cluster peaked at the left uncus, Brodmann area (BA) 20, and one at the middle cingulate gyrus (MCG), BA 24. The remaining regions were in the right hemisphere, where one region peaked at the middle occipital gyrus (MOG) (BA 19), one region at the superior temporal gyrus (STG) (BA 41) and one cluster with two peaks at the middle temporal gyrus (MTG) and the Inferior Temporal Gyrus (BA 41 and 39 respectively). The resulting map overlays were produced on a standardised structural scan using Mango v4.1 [104] (rii.uthscsa.edu/mango).

**Table 6:** Regions with significantly elevated activation likelihood from the ALE analysis

Comparison	Cluster	Brain region		BA	Volume (mm^3^)	Talairach			ALE (10^−2^)	Range						Centred at		
						x	y	z		From			To					
										x	y	z	x	y	z	x	y	z
NT > ASD	#1	R	STG	41	408	44	− 32	4	1.69	40	− 34	0	48	− 28	6	44.1	− 31.3	3.1
	#2	R	MTG	39	312	48	− 60	8	1.07	46	− 64	4	50	− 56	12	49	− 62.2	5.1
			ITG			50	− 68	0										
	#3	R	MOG	19	264	46	− 74	− 6	1.20	40	− 76	− 8	48	− 70	− 4	44.4	− 72.7	− 6.2
	#4	L	Uncus	20	248	− 32	− 4	− 28	1.21	− 36	− 8	− 30	− 30	0	− 26	− 32.7	− 4.3	− 28.1
	#5	L	MCG	24	408	− 8	− 4	46	1.72	− 12	− 8	42	− 4	0	50	− 8	− 4.5	45.7

*BA* Brodmann area, *STG* superior temporal gyrus, *MTG* middle temporal gyrus, *ITG* inferior temporal gyrus, *MOG* middle occipital gyrus, *MCG* middle cingulate gyrus, *R* right, *L* left

## Discussion

The aim of this meta-analysis was to investigate whether ASD individuals show differences in their ability to perceive and interpret biological motion when compared to NT individuals. This question has been under discussion for decades and contradicting results have continuously appeared in the literature. Therefore, a quantitative summary of the results was necessary to allow research to move forward in understanding the atypicalities present in ASD. The current study investigated several potential factors that could contribute to the variable and often mixed results in this field. We explored the possibility of different paradigms being a reason for these varied findings and the effect of age, sex and IQ on participants’ performance.

This meta-analysis showed that there is a medium effect indicating an overall decreased performance in perceiving and interpreting biological motion for ASD individuals. Specifically, the present findings show that individuals with autism show lower levels of performance when higher order information, such as emotion, is required to be extracted from biological motion. Moreover, age is a significant contributing factor to the variability of the results, as different age groups show different degrees of performance decrement. Additionally, we did not find a significant effect in reaction time data, suggesting no delays responding to stimuli once recognised. Further, the effect size of the eye-tracking results would argue that autistic individuals do not attend to or orient towards BM. However, the small sample of studies and its variability lead to a non-significant estimated effect size, even though the effect size would be constituted as ‘large’. This variability is evident in the distribution of the study effect sizes around the average effect size. Thus, the absence of significance in the eye-tracking results may possibly be mainly attributed to the small sample. A similar pattern is seen from the EEG studies. Finally, the five clusters identified in the fMRI ALE analysis to show higher activation for NT than ASD individuals provide evidence for a potential neural basis for the difference in BM perception abilities.

### Differences in performance increase with the increase in task complexity

Biological motion can convey various types of information. It can provide simple information about what others around us are doing, or more complex information, for example about the emotional state of others [1, 2]. All this information is of great importance in social interaction. Although, Koldewyn et al. [22] argue that individuals with ASD can perceive/detect biological motion, we found a general decreased performance in the perception of BM in ASD individuals in all paradigms, including simple BM detection. Moreover, there was no difference in performance between BM detection and action recognition. This indicates that although biological motion detection requires simple integration of motion elements, decreased performance at this level already exists, hindering recognition. Furthermore, the effect size of the difference between the NT and ASD individuals was about twice the size when emotion recognition paradigms were employed. Thus, aligned with Koldewyn et al.’s [22] arguments, there is in fact decreased performance when the extraction of emotion information is required but this would manifest on top of the already existing decreased performance with simple detection of BM. Similar findings were also observed by Federici et al. (41), where inferring higher order information from PLDs showed larger effects. This is an expected finding since ASD is defined with difficulties in social interaction and communication. Emotion recognition is a highly social process, making it more cognitively demanding than BM identification which would rely on perceptual decisions. The effect of paradigm in our meta-analysis may be because emotion adds an additional layer of social complexity in comparison to simple BM identification or action recognition, making it more difficult for individuals with ASD to perform on such tasks. This difference between the two groups is true even when simple and complex emotional recognition tasks are used ([23, 105–107], but see [108]).

It is worth noting that we did not find significant effects when reaction time was the measured outcome. Even more, the effect size that we found would be considered small according to Cohen’s [97] characterisations. Although, a recent meta-analysis has shown that global information integration takes time in autism, which is evident in slower reaction times [21], this is not evident in biological motion perception. A possible explanation is that motion introduces an additional factor, which is suggested by reported higher motion thresholds in autism [13, 109]. Moreover, biological motion perception has longer spatiotemporal integration windows than simple motion stimuli, which could make it more difficult to detect small differences in reaction time [110]. Thus, the decreased performance in perceiving biological motion is a combination between motion and the social factor of human movement, which is more evident in interpretation, rather than in time taken for processing.

This finding, that different paradigms introduce varying effect sizes emphasises that when the research community is trying to explain differences between NT and ASD individuals, it cannot simply talk about biological motion perception as a whole. Instead, the nuances that different paradigms bring need to be emphasised. Moreover, the different paradigms are not comparable; instead they provide different levels of understanding of the abilities of individuals with ASD.

### Differences between ASD and NT individuals decrease with age

The developmental course of BM perception in ASD is critically important, especially since so many contradicting results have been found between different age groups [12, 14, 46, 49, 60, 64]. Overall, it appears that the size of the difference between the two groups is larger when children are investigated. On the other hand, the effect size when adults were studied did not differ from the effect size when adolescents were studied.

Our findings imply that ASD individuals tend to catch up with age and that performance within ASD becomes more aligned with the NT population. This in turn corresponds to the general improvement with age observed within NT individuals [111]. Despite this catch up however, the size of the differences between the two groups was significant at every age category, indicating consistent difference in performance but to a varying degree dependent on age. Thus, whilst NT and ASD tend to both improve in their ability to detect BM, ASD individuals do so at a slower rate. This implies the existence of a developmental delay in the extraction of relevant social information from biological motion. It should be noted that Annaz et al. [13] also did not find a relationship with age in children with ASD for non-biological motion coherence and form-from-motion paradigms, whereas the effect was present in NT individuals. Thus, it appears that there might be a global delay in motion coherence sensitivity in ASD. Although, Simmons et al. [7] argue for inconsistency in the literature about motion coherence and ASD, elevated motion coherence thresholds have been found by others (e.g. [19, 22]). Moreover, Van der Hallen et al.’s [40] findings suggest specifically that there is an overall decreased performance in global motion perception in individuals with ASD, for both coherent and biological motion.

To sum, the variability in the behavioural findings in the literature can be explained largely by the fact that ASD participants cannot be put together as a single group. As well as talking about the nuances that individual paradigms bring, we need to distinguish between the different age groups. Thus, a study aiming to investigate performance in adults should not look for effects as large as the ones found in children, as they are statistically not comparable.

### No effect of sex, FSIQ and NVIQ on performance on BM paradigms

It has been suggested that ASD is expressed differently in males and females and that females could be the source of variability in some of the results related to performance in the ASD literature [21]. However, we did not find any significant effects of the proportion of females in either the NT or ASD sample. Furthermore, neither the FSIQ nor the NVIQ of either group revealed a significant effect on the overall performance. Although some studies have argued for [17, 18] and against [19, 20, 40] the effects of IQ, those that find effects usually have lower IQ scores in comparison to the ones that do not find this effect (but see ref [10]). The mean FSIQ in the current meta-analysis was also higher—with averages in the behavioural, eye-tracking and fMRI designs falling between 103 and 112. Thus, it is possible that any variability that may be explained from an IQ perspective might not have been captured in this analysis or in studies where the IQs are above 100. Thus, the present findings may not necessarily be transferable to ASD individuals at the lower end of the IQ distribution. However, since research is usually done on individuals of average or above average IQ, this nuance would not be captured unless more research is adapted and done with individuals on the lower side of the IQ distribution.

### Brain and behaviour

From a brain imaging perspective, we aimed to investigate both EEG and fMRI. This was driven by the fact that it has been suggested that individuals with ASD utilise different brain networks when observing biological motion [14].

EEG studies, which usually rely on mu-suppression as a proxy for the MNN in ASD, argue for an impaired mirror system in autism [35, 38, 67, 112]. Specifically, they have consistently found reduced mu-suppression in central electrodes. Similar findings have been indicated by a meta-analysis conducted by Fox et al. [37]. However, we did not find a significant effect for the difference between ASD and NT individuals. There are two possible explanations for this result. One possibility is that the effect sizes were too small to be considered significantly different from 0. This, however, does not seem to be the case, as there is a good distribution of results on both sides of the no-difference line. The second possibility is that the small sample of studies did not provide enough data points to allow for a stable estimate to be given. This is especially evident by the lower bound of the 95% CI for the overall effect size, as it stays very slightly below 0. Furthermore, the exploratory analysis, which is reported in Additional file 3, showed that depending on the frequency used to perform the analysis, the effect size can differ greatly. Thus, for some conclusion to be made from the EEG studies, a common analysis structure needs to be agreed upon. However, Hamilton [43] argues that support for a difference from these studies is weak and mixed, which also speaks for the unreliable findings. Moreover, it has been argued that mu suppression findings can be unreliable as they are very much dependent on the baseline that is chosen [113]. Although some of the studies identified here used the same paradigm with the same baseline [35, 112, 114], this was not the case for all of them [38, 67], which makes it difficult to compare the findings. Thus, a general standard for data analysis and what constitutes as a baseline needs to be set before any conclusions can be drawn.

From an fMRI perspective, we investigated the differences in brain activation between ASD and NT in biological motion perception and emotion recognition. It is noteworthy that emotion perception and BM observation paradigms were analysed together, due to the small sample size. Unfortunately, we were unable to identify significant clusters that overlapped between the studies. However, the exploratory analysis showed that by using a more relaxed threshold, the areas that come up as different between the two groups correspond to the areas that have been identified in the biological motion perception literature.

In short, we found five clusters where NT individuals showed greater activation than ASD individuals: the left uncus, left middle cingulate gyrus, right middle occipital gyrus and one cluster peaking at the right superior and middle temporal gyri. These findings are consistent with literature showing right hemisphere dominance in the processing of biological motion [115, 116]. Particularly, the right ITG and the right middle temporal gyrus (MTG) have been observed to be specifically implicated in the observation of human motion [116–118]. Additionally, the ITG has been found to be part of the BM processing network of NT in McKay et al.’s [14] experiment but not in ASD, which corresponds to our findings. Similarly, the MTG is related to the perception of human movement. Peelen and Downing [119] argue that the MTG is part of the extrastriate body area (EBA) and that its activation during action observation is due to it representing the shape and posture of the body rather than the action. Additionally, Thompson and Baccus [120] argue that motion and form make independent contributions to the processing of biological motion in the MT areas. Specifically, the MT areas respond a lot more to the motion aspects, and EBA to the representation of human form. However, since these areas overlap [120] and the observed cluster in these results peaked at MTG and ITG, it could be expected that the activation is due to an interplay between the motion and human form information. This collaborative mechanism has previously been suggested by Downing and Peelen [115]. If individuals with ASD have problems perceiving the basic human shape and posture, it is understandable why there appeared to be consistent differences in behavioural performance between ASD and NT individuals in all biological motion paradigms investigated here. Moreover, as mentioned earlier, with the increased motion thresholds found within individuals with ASD [109], it could be expected that impairments would come from both motion and human form detection.

Interestingly, the superior temporal sulcus (STS) is a region that has been implied to be important in biological motion perception [2, 116]; however, we did not find higher STS activation in NT in comparison to ASD. Nevertheless, we did find the superior temporal gyrus (STG) to have higher activation in NT. Previous findings [2, 116, 121] have argued that the STS is involved in social perception, namely it integrates the social context with the actor’s actions. Nevertheless, McKay et al. [14] also did not find the STS to be involved in simple biological motion perception. Since their paradigm is similar to the paradigms used in the papers, which dominated in the present analysis, it fits that we also did not find STS activation. However, the proximity of the STG to the STS suggests that there might be some potential overlap which could be driven by the inclusion of the emotion-related BM paradigms in the analysis. In fact, the STG has been found to show activation when observing emotional biological motion and in biological motion perception paradigms in general [116, 122, 123].

Despite both the low number of studies which were included in the ALE analysis and the exploratory nature of the results, the brain areas found were consistent with BM processing literature. Moreover, differences in these brain areas can and do show differences in behaviour. This finding emphasises the connection between brain differences and behavioural performance. However, due to the small number of studies and the fact that a more constrained threshold did not show any significant values, some caution needs to be taken when interpreting these results.

### Methodological limitations

The quality of a meta-analysis is only as high as the quality of the studies that it includes. The studies that we included received a relatively high score on our quality assessment measure with little variance between the studies. The major methodological issues of the included studies were the small sample sizes and the fact that on several occasions there were no corrections for multiple comparisons. However, the correction for multiple comparisons should not have affected our results as we used the descriptive or test statistics, rather than the *p* values. Nevertheless, it was evident in the behavioural analysis that the quality of the studies played a significant role in reducing variability and allowing for better interpretability of the statistical results. This indicates that small changes in the quality of a study contributed enough to influence the results. Specifically, it appeared that the higher the quality of a study, the smaller the effect size was; indicating that better controlled studies produced smaller effect sizes. The same finding was observed by the publication bias analysis, which showed that studies with smaller standard errors produced smaller effect sizes. This on its own is an important discovery about the control that is used when developing a study paradigm. It is possible that with a better controlled study, larger amounts of variability are controlled, reducing any additional external effects. Thus, future autism researchers should aim to provide even more methodologically sound results, to allow them to distinguish between external heterogeneity and within-ASD heterogeneity.

Additionally, in our criteria ,we aimed to include studies that utilised either the gold standard (i.e. ADOS plus ADI; see [7]) or expert clinical opinion when confirming the ASD diagnosis of their participants. However, during the selection process, we realised that a number of studies did not employ the gold standard and rather used various diagnostic measures. For that reason, we expanded our inclusion criteria to include at least some form of diagnosis confirmation. Worryingly, one of the reasons that studies were not included in the present analysis was that the diagnosis was not confirmed by any means, let alone by using the gold standard. However, the concept of a gold standard is a matter of debate [124] and it has been noted that the scales do not always capture individuals that have been diagnosed with Asperger’s syndrome [45]. Thus, how ASD participants ought to be identified in future studies needs to be explored.

Furthermore, even though it is argued that a quantitative summary on two effect sizes is better than simple counts of positive vs. negative effects [125], statistical analysis, and the confidence one can give to it, is proportionally dependent to its sample size. Although the three-level model has allowed us to utilise more than one effect size per study, thus increasing the number of cases included, the resulting sample is still small, especially for some of the categories of analysis. This is mainly true for the EEG analysis, where one study provided most of the effect sizes. Thus, when interpreting the results from this meta-analysis, the number of studies in each part needs to be considered. Furthermore, the number of effect sizes that we were able to include in some of the analyses (eye-tracking, RT, EEG and fMRI) did not allow us to investigate important factors such as paradigm and age. This unfortunately limits our ability to interpret the effect of those factors. Nevertheless, if we look at the behavioural results, then we can conjecture that these factors will be important and will also need to be considered, when new paradigm designs are considered, or when interpreting the overall weight of the effects found in the literature.

Finally, we included studies from unpublished sources, such as dissertations and theses in an attempt to reduce the chances of a publication bias. Nevertheless, most of these unpublished sources were significant. However, this does not exclude the ‘file drawer effect’ where non-significant findings are likely to not be published. It is also possible that the Egger regression method is capturing other types of bias, for example the heterogeneity between the studies themselves, which is expected due to the ASD population being heterogeneous [102].

## Conclusions and future directions

Overall, it appears that individuals with ASD show lower performance measures than NT individuals on tasks involving the detection and interpretation of BM. However, age and the type of paradigm used have a great influence on the size of the difference between ASD individuals’ performance and the performance of NT individuals. We show that there is a developmental delay in BM understanding, which improves with age within the ASD population and explains the high variability in the results established in the literature. Moreover, autistic individuals show consistently lower performance in paradigms requiring the extraction of emotion from BM in comparison to action recognition or simple BM detection. This finding is more meaningful, considering that a main characteristic of ASD is an impairment in social communication and that interaction and emotional portrayal of biological motion has great social relevance. Finally, we find that there appear to be differences between ASD and NT groups in brain activations when viewing BM and those differences can provide an insight to why the behaviour that we observe exists.

For the field of research to move forward, methodological standards need to be imposed in terms of the age ranges incorporated, and the types of paradigms used. However, interpretation standards need to be considered as well. Although it appears that there is variability in the literature as to whether and how large the effects are, the effects are actually varied due to the combination of various factors. For proper interpretation of the field, the paradigm used and the age of the participants need to be considered as segregating factors. This is important because a child with autism might have difficulty perceiving biological motion, but by the time they reach adulthood, that effect might have subsided. Similarly, individuals with autism might find it much more difficult to extract emotion information from human movement, but they are much better at describing non-affective actions. Finally, as a field, autism research is going to find heterogeneous findings, due to the innate variability between autistic individuals. However, sound methodological principles when developing studies will reduce that variability and allow for better consistency and easier interpretation.

## Supplementary information

**Additional file 1.** Search strategies and number of records per database. File includes the search strategies used for the extraction of the papers from the five electronic databases mentioned in text and a table with the number of records extracted from each.

**Additional file 2.** Weight of Evidence analysis. File includes the weight of evidence criteria for scoring and the summary statistics for Weight of Evidence scores.

**Additional file 3.** Exploratory EEG analysis. File includes an exploratory analysis of the EEG data with frequency as a predictive factor, as refered to from the text.

## Data Availability

The dataset(s) supporting the conclusions of this article are available in the ReShare repository. 10.5255/UKDA-SN-853277.

## Abbreviations

(med)SFG: (medial)Superior frontal gyrus
(p)STS: (posterior) Superior temporal sulcus
ACG: Anterior cingulate gyrus
AG: Angular gyrus
AR: Action recognition
ASD: Autism spectrum disorder
BA: Broadman area
BM: Biological motion
COH: Coherent dot motion
D: Biological motion detection
EEG: Electroencephalogram
ER: Emotion recognition
FFG: Fusiform gyrus
FLD: Full-light display
fMRI: Functional magnetic resonance imaging
FSIQ: Full-scale intelligence quotient
*g*: Hedges’ *g*
IFG: Inferior frontal gyrus
IOG: Inferior occipital gyrus
IPL: Inferior parietal lobule
ITG: Inferior temporal gyrus
L: Left
MCG: Middle cingulate gyrus
MFG: Middle frontal gyrus
MNN: Mirror neuron network
MOG: Middle occipital gyrus
MT: Middle temporal area
MTG: Middle temporal gyrus
N: Sample size
NT: Neurotypically developing
NVIQ: Non-verbal intelligence quotient
PABAK: Prevalence-adjusted and bias-adjusted kappa
PLD: Point-light display
PTS: Posterior temporal sulcus
PV: Passive viewing
R: Right
RT: Reaction time
SCR: Scrambled biological motion
SE(*g*): Estimated standard error of *g*
SQA: Standard quality assessment score
STG: Superior temporal gyrus
TPJ: Temporal-parietal junction
var. *g*: Estimated variance of *g*
vlPC: Ventrolateral prefrontal cortex
vmPC: Ventromedial prefrontal cortex
WB: Whole brain analysis
WoE: Weight of evidence

## References

[1] Blake R, Shiffrar M (2007). Perception of human motion. Annu Rev Psychol..

[2] Pavlova MA (2012). Biological Motion processing as a hallmark of social cognition. Cereb Cortex May..

[3] Pollick FE, Paterson HM, Bruderlin A, Sanford AJ (2001). Perceiving affect from arm movement. Cognition..

[4] Johansson G (1973). Visual perception of biological motion and a model for its analysis. Percept Psychophys..

[5] Klin A, Lin DJ, Gorrindo P, Ramsay G, Jones W (2009). Two-year-olds with autism orient to non-social contingencies rather than biological motion. Nature..

[6] Sparrow WA, Shinkfield AJ, Day RH, Zerman L (1999). Visual perception of human activity and gender in biological-motion displays by individuals with mental retardation. Am J Ment Retard..

[7] Simmons DR, Robertson AE, McKay LS, Toal E, McAleer P, Pollick FE (2009). Vision in autism spectrum disorders. Vision Res..

[8] American Psychiatric Association., American Psychiatric Association. DSM-5 Task Force. Diagnostic and statistical manual of mental disorders : DSM-5. p. 947.

[9] Hubert B, Wicker B, Moore DG, Monfardini E, Duverger H, Da Fonséca D (2007). Brief report: recognition of emotional and non-emotional biological motion in individuals with autistic spectrum disorders. J Autism Dev Disord..

[10] Murphy P, Brady N, Fitzgerald M, Troje NF (2009). No evidence for impaired perception of biological motion in adults with autistic spectrum disorders. Neuropsychologia..

[11] Saygin AP, Cook J, Blakemore S-JJ (2010). Unaffected perceptual thresholds for biological and non-biological form-from-motion perception in autism spectrum conditions. PLoS One..

[12] Parron C, De Fonseca D, Santos A, Monfardini E, Deruelle C, Da Fonseca D (2008). Recognition of biological motion in children with autistic spectrum disorders. Autism..

[13] Annaz D, Remington A, Milne E, Coleman M, Campbell R, Thomas MSC (2010). Development of motion processing in children with autism. Dev Sci..

[14] McKay LS, Simmons DR, McAleer P, Marjoram D, Piggot J, Pollick FE (2012). Do distinct atypical cortical networks process biological motion information in adults with autism spectrum disorders?. Neuroimage..

[15] Nackaerts E, Wagemans J, Helsen W, Swinnen SP, Wenderoth N, Alaerts K (2012). Recognizing biological motion and emotions from point-light displays in autism spectrum disorders. PLoS One..

[16] Blake R, Turner LMM, Smoski MJJ, Pozdol SLL, Stone W (2003). L. Visual recognition of biological motion is impaired in children with autism. Psychol Sci..

[17] Jones CRG, Swettenham J, Charman T, Marsden AJS, Tregay J, Baird G (2011). No evidence for a fundamental visual motion processing deficit in adolescents with autism spectrum disorders. Autism Res..

[18] Rutherford MD, Troje NF (2012). IQ predicts biological motion perception in autism spectrum disorders. J Autism Dev Disord..

[19] Atkinson AP (2009). Impaired recognition of emotions from body movements is associated with elevated motion coherence thresholds in autism spectrum disorders. Neuropsychologia..

[20] Van Boxtel JJA, Dapretto M, Lu H (2016). Intact recognition, but attenuated adaptation, for biological motion in youth with autism spectrum disorder. Autism Res..

[21] Van der Hallen R, Evers K, Brewaeys K, Van den Noortgate W, Wagemans J (2015). Global processing takes time: A meta-analysis on local–global visual processing in ASD. Psychol Bull..

[22] Koldewyn K, Whitney D, Rivera SM (2010). The psychophysics of visual motion and global form processing in autism. Brain..

[23] Fridenson-Hayo S, Berggren S, Lassalle A, Tal S, Pigat D, Bölte S (2016). Basic and complex emotion recognition in children with autism: cross-cultural findings. Mol Autism..

[24] Chita-Tegmark M (2016). Social attention in ASD: a review and meta-analysis of eye-tracking studies. Res Dev Disabil..

[25] de Gelder B (2009). Why bodies? Twelve reasons for including bodily expressions in affective neuroscience. Philos Trans R Soc Lond B Biol Sci..

[26] Annaz D, Campbell R, Coleman M, Milne E, Swettenham J (2012). Young children with autism spectrum disorder do not preferentially attend to biological motion. J Autism Dev Disord..

[27] Falck-Ytter T, Rehnberg E, Bö Lte S, Bölte S (2013). Lack of visual orienting to biological motion and audiovisual synchrony in 3-year-olds with autism. PLoS One..

[28] Fujioka T, Inohara K, Okamoto Y, Masuya Y, Ishitobi M, Saito DN (2016). Gazefinder as a clinical supplementary tool for discriminating between autism spectrum disorder and typical development in male adolescents and adults. Mol Autism..

[29] Fujisawa TX, Tanaka S, Saito DN, Kosaka H, Tomoda A (2014). Visual attention for social information and salivary oxytocin levels in preschool children with autism spectrum disorders: an eye-tracking study. Front Neurosci..

[30] Alaerts K, Swinnen SP, Wenderoth N (2017). Neural processing of biological motion in autism: an investigation of brain activity and effective connectivity. Sci Rep..

[31] Freitag CM, Konrad C, Häberlen M, Kleser C, Von Gontard A, Reith W (2008). Perception of biological motion in autism spectrum disorders. Neuropsychologia..

[32] Grèzes J, Wicker B, Berthoz S, de Gelder B (2009). A failure to grasp the affective meaning of actions in autism spectrum disorder subjects. Neuropsychologia..

[33] Kaiser MD, Shiffrar M (2009). The visual perception of motion by observers with autism spectrum disorders: a review and synthesis. Psychon Bull Rev..

[34] Villalobos ME, Mizuno A, Dahl BC, Kemmotsu N, Müller R-A (2005). Reduced functional connectivity between V1 and inferior frontal cortex associated with visuomotor performance in autism. Neuroimage..

[35] Oberman LM, Hubbard EM, McCleery JP, Altschuler EL, Ramachandran VS, Pineda JA (2005). EEG evidence for mirror neuron dysfunction in autism spectrum disorders. Cogn Brain Res..

[36] Williams JHG, Waiter GD, Gilchrist A, Perrett DI, Murray AD, Whiten A (2006). Neural mechanisms of imitation and “mirror neuron” functioning in autistic spectrum disorder. Neuropsychologia..

[37] Fox NA, Bakermans-Kranenburg MJ, Yoo KH, Bowman LC, Cannon EN, Vanderwert RE (2016). Assessing human mirror activity with EEG mu rhythm: a meta-analysis. Psychol Bull..

[38] Raymaekers R, Wiersema JR, Roeyers H (2009). EEG study of the mirror neuron system in children with high functioning autism. Brain Res..

[39] Kaiser MD, Pelphrey KA (2012). Disrupted action perception in autism: behavioral evidence, neuroendophenotypes, and diagnostic utility. Dev Cogn Neurosci..

[40] Van der Hallen R, Manning C, Evers K, Wagemans J (2019). Global motion perception in autism spectrum disorder: a meta-analysis. J Autism Dev Disord..

[41] Federici A, Parma V, Vicovaro M, Radassao L, Casartelli L, Ronconi L. Anomalous perception of biological motion in autism: a conceptual review and meta-analysis. bioRxiv. 2019. 10.1038/s41598-020-61252-3 PMC7067769 32165647

[42] Philip RCMM, Dauvermann MR, Whalley HC, Baynham K, Lawrie SM, Stanfield AC (2012). A systematic review and meta-analysis of the fMRI investigation of autism spectrum disorders. Neurosci Biobehav Rev..

[43] Hamilton AF (2013). Reflecting on the mirror neuron system in autism: a systematic review of current theories. Dev Cogn Neurosci..

[44] Moher D, Shamseer L, Clarke M, Ghersi D, Liberati A, Petticrew M (2015). Preferred reporting items for systematic review and meta-analysis protocols (PRISMA-P) 2015 statement. Syst Rev..

[45] Price KJ, Shiffrar M, Kerns KA (2012). Movement perception and movement production in Asperger’s Syndrome. Res Autism Spectr Disord..

[46] Wang L-H, Chien SHL, Hu S-F, Chen T-Y, Chen H-S, Hui S (2015). Children with autism spectrum disorders are less proficient in action identification and lacking a preference for upright point-light biological motion displays. Res Autism Spectr Disord..

[47] Byrt T, Bishop J, Carlin JB (1993). Bias, prevalence and kappa. J Clin Epidemiol..

[48] Philip RCM, Whalley HC, Stanfield AC, Sprengelmeyer R, Santos IM, Young AW (2010). Deficits in facial, body movement and vocal emotional processing in autism spectrum disorders. Psychol Med..

[49] Actis-Grosso R, Bossi F, Ricciardelli P (2015). Emotion recognition through static faces and moving bodies: a comparison between typically developed adults and individuals with high level of autistic traits. Front Psychol..

[50] Alaerts K, Geerlings F, Herremans L, Swinnen SP, Verhoeven J, Sunaert S (2015). Functional organization of the action observation network in autism: a graph theory approach. PLoS One..

[51] Binnersley J. Perception of biological motion in autistic spectrum disorder: University College London; 2006.

[52] Cook J, Saygin AP, Swain R, Blakemore S-JJ (2009). Reduced sensitivity to minimum-jerk biological motion in autism spectrum conditions. Neuropsychologia..

[53] Couture SM, Penn DL, Losh M, Adolphs R, Hurley R, Piven J (2010). Comparison of social cognitive functioning in schizophrenia and high functioning autism: more convergence than divergence. Psychol Med..

[54] Cusack JP, Williams JHG, Neri P (2015). Action perception is intact in autism spectrum disorder. J Neurosci..

[55] Edey R, Cook J, Brewer R, Bird G, Press C (2019). Adults with autism spectrum disorder are sensitive to the kinematic features defining natural human motion. Autism Res..

[56] Karuppali S (2018). Do children with autism spectrum disorders exhibit biological motion perception deficits? Evidence using an action recognition paradigm. J Indian Assoc Child Adolesc Ment Heal..

[57] Koldewyn K, Whitney D, Rivera SM (2011). Neural correlates of coherent and biological motion perception in autism. Dev Sci..

[58] Krakowski A (2014). Biological motion processing in typical development and in the autism spectrum.

[59] Kröger A, Bletsch A, Krick C, Siniatchkin M, Jarczok TA, Freitag CM (2014). Visual event-related potentials to biological motion stimuli in autism spectrum disorders. Soc Cogn Affect Neurosci..

[60] Krüger Britta, Kaletsch Morten, Pilgramm Sebastian, Schwippert Sven-Sören, Hennig Jürgen, Stark Rudolf, Lis Stefanie, Gallhofer Bernd, Sammer Gebhard, Zentgraf Karen, Munzert Jörn (2017). Perceived Intensity of Emotional Point–Light Displays is Reduced in Subjects with ASD. Journal of Autism and Developmental Disorders.

[61] Morrison KE, Pinkham AE, Kelsven S, Ludwig K, Penn DL, Sasson NJ (2019). Psychometric evaluation of social cognitive measures for adults with autism. Autism Res..

[62] Sotoodeh MS, Taheri-Torbati H, Sohrabi M, Ghoshuni M (2019). Perception of biological motions is preserved in people with autism spectrum disorder: electrophysiological and behavioural evidences. J Intellect Disabil Res..

[63] Swettenham J, Remington A, Laing K, Fletcher R, Coleman M, Gomez J-CC (2013). Perception of pointing from biological motion point-light displays in typically developing children and children with autism spectrum disorder. J Autism Dev Disord..

[64] Turi M, Muratori F, Tinelli F, Morrone MC, Burr DC (2017). Autism is associated with reduced ability to interpret grasping actions of others. Sci Rep..

[65] Von Der Lühe T, Manera V, Barisic I, Becchio C, Vogeley K, Schilbach L (2016). Interpersonal predictive coding, not action perception, is impaired in autism. Philos Trans R Soc..

[66] Burnside K, Wright K, Poulin-Dubois D (2017). Social motivation and implicit theory of mind in children with autism spectrum disorder. Autism Res..

[67] Bernier R, Dawson G, Webb S, Murias M (2007). EEG mu rhythm and imitation impairments in individuals with autism spectrum disorder.

[68] Bernier R, Aaronson B, Mcpartland J (2013). The role of imitation in the observed heterogeneity in EEG mu rhythm in autism and typical development. Brain Cogn..

[69] Hirai M, Gunji A, Inoue Y, Kita Y, Hayashi T, Nishimaki K (2014). Differential electrophysiological responses to biological motion in children and adults with and without autism spectrum disorders. Res Autism Spectr Disord..

[70] Dumas G, Soussignan R, Hugueville L, Martinerie J, Nadel J (2014). Revisiting mu suppression in autism spectrum disorder. Brain Res..

[71] Alaerts K, Woolley DG, Steyaert J, Di Martino A, Swinnen SP, Wenderoth N (2013). Underconnectivity of the superior temporal sulcus predicts emotion recognition deficits in autism. Soc Cogn Affect Neurosci..

[72] Björnsdotter M, Wang N, Pelphrey K, Kaiser MD (2016). Evaluation of quantified social perception circuit activity as a neurobiological marker of autism spectrum disorder. JAMA psychiatry..

[73] Jack A, Morris JP (2014). Neocerebellar contributions to social perception in adolescents with autism spectrum disorder. Dev Cogn Neurosci..

[74] Jack A, Keifer CM, Pelphrey KA (2017). Cerebellar contributions to biological motion perception in autism and typical development. Hum Brain Mapp..

[75] Kaiser MD, Hudac CM, Shultz S, Lee SM, Cheung C, Berken AM (2010). Neural signatures of autism. PNAS..

[76] Marsh LE, Hamilton AF de C. (2011). Dissociation of mirroring and mentalising systems in autism. Neuroimage..

[77] Yang YJD, Sukhodolsky DG, Lei J, Dayan E, Pelphrey KA, Ventola P. Distinct neural bases of disruptive behavior and autism symptom severity in boys with autism spectrum disorder. J Neurodev Disord. 2017;9(1). 10.1186/s11689-017-9183-z PMC5240249 28115995

[78] Kmet LM, Lee RC, Cook LS. Standard quality assessment criteria for evaluating primary research papers from a variety of fields: University of Alberta Libraries; 2004.

[79] Gough D (2007). Weight of Evidence: a framework for the appraisal of the quality and relevance of evidence. Res Pap Educ..

[80] Del Re AC. compute.es: Compute Effect Sizes. R package. 2013.

[81] R Core Team (2018). R: A language and environment for statistical computing.

[82] RStudio Team (2016). RStudio: Integrated Development for R.

[83] Cheung MW-L (2014). Modeling dependent effect sizes with three-level meta-analyses: a structural equation modeling approach. Psychol Methods..

[84] Van den Noortgate W, López-López JA, Marín-Martínez F, Sánchez-Meca J (2013). Three-level meta-analysis of dependent effect sizes. Behav Res Methods..

[85] Van den Noortgate W, López-López JA, Marín-Martínez F, Sánchez-Meca J (2015). Meta-analysis of multiple outcomes: a multilevel approach. Behav Res Methods..

[86] SAS OnDemand for Academics: SAS Studio. Cary: SAS Institute Inc;

[87] Schaalje GB, McBride JB, Fellingham GW. Approximations to distributions of test statistics in complex mixed linear models using SAS Proc MIXED. In: SAS Conference Proceedings, vol. 26: SAS Users Group International; 2001. p. 262.

[88] Higgins JPT, Thompson SG (2002). Quantifying heterogeneity in a meta-analysis. Stat Med..

[89] Egger M, Davey Smith G, Schneider M, Minder C (1997). Bias in meta-analysis detected by a simple, graphical test. BMJ..

[90] Duval S, Tweedie R (2000). Trim and fill: a simple funnel-plot-based method of testing and adjusting for publication bias in meta-analysis. Biometrics..

[91] Rendina-Gobioff G, Kromery JD. PUB_BIAS: a SAS macro for detecting publication bias in meta-analysis. In: 14th Annual SouthEast SAS Users Group (SESUG) Conference. Atlanta; 2006.

[92] Turkeltaub PE, Eickhoff SB, Laird AR, Fox M, Wiener M, Fox P (2012). Minimizing within-experiment and within-group effects in activation likelihood estimation meta-analyses. Hum Brain Mapp..

[93] Eickhoff SB, Laird AR, Grefkes C, Wang LE, Zilles K, Fox PT (2009). Coordinate-based activation likelihood estimation meta-analysis of neuroimaging data: a random-effects approach based on empirical estimates of spatial uncertainty. Hum Brain Mapp..

[94] Eickhoff SB, Bzdok D, Laird AR, Kurth F, Fox PT (2012). Activation likelihood estimation meta-analysis revisited. Neuroimage..

[95] Ouzzani M, Hammady H, Fedorowicz Z, Elmagarmid A (2016). Rayyan—a web and mobile app for systematic reviews. Syst Rev..

[96] Moher D, Liberati A, Tetzlaff J, Altman DG (2009). Preferred reporting items for systematic reviews and meta-analyses: the PRISMA statement. PLoS Med..

[97] Cohen J (1988). Statistical power analysis for the behavioral sciences.

[98] Nackaerts Evelien, Wagemans Johan, Helsen Werner, Swinnen Stephan P., Wenderoth Nicole, Alaerts Kaat (2012). Recognizing Biological Motion and Emotions from Point-Light Displays in Autism Spectrum Disorders. PLoS ONE.

[99] Scheepers C (2014). Between-group matching of confounding variables: why covariates remain important for analysis.

[100] Price KJ, Shiffrar M, Kerns KA. Movement perception and movement production in Asperger’s syndrome previously researchers have documented movement impairments in individuals with Asperger’s Syndrome (AS) and autism (e.g. Res Autism Spectr Disord. 2011;6:391–398.

[101] Sterne JA, Egger M (2001). Funnel plots for detecting bias in meta-analysis. J Clin Epidemiol..

[102] Sterne JAC, Sutton AJ, Ioannidis JPA, Terrin N, Jones DR, Lau J (2011). Recommendations for examining and interpreting funnel plot asymmetry in meta-analyses of randomised controlled trials. BMJ.

[103] Higgins J, Green J. Cochrane Handbook for Systematic Reviews of Interventions: The Cochrane Collaboration; 2011.

[104] Lancaster JL, Martinez MJ. Mango: Multi-image Analysis GUI.

[105] Bal E, Harden E, Lamb D, Van Hecke AV, Denver JW, Porges SW (2010). Emotion recognition in Children with autism spectrum disorders: relations to eye gaze and autonomic state. J Autism Dev Disord..

[106] Dyck MJ, Ferguson K, Shochet IM (2001). Do autism spectrum disorders differ from each other and from non-spectrum disorders on emotion recognition tests?. Eur Child Adolesc Psychiatry..

[107] Hudepohl MB, Robins DL, King TZ, Henrich CC (2015). The role of emotion perception in adaptive functioning of people with autism spectrum disorders. Autism..

[108] Jones CRG, Pickles A, Falcaro M, Marsden AJS, Happé F, Scott SK (2011). A multimodal approach to emotion recognition ability in autism spectrum disorders. J Child Psychol Psychiatry..

[109] Pellicano E, Gibson L, Maybery M, Durkin K, Badcock DR (2005). Abnormal global processing along the dorsal visual pathway in autism: A possible mechanism for weak visuospatial coherence?. Neuropsychologia..

[110] Neri P, Morrone MC, Burr DC (1998). Seeing biological motion. Nature..

[111] Ghanouni P, Memari AH, Shayestehfar M, Moshayedi P, Gharibzadeh S, Ziaee V (2015). Biological motion perception is affected by age and cognitive style in children aged 8–15. Neurol Res Int..

[112] Oberman LM, McCleery JP, Hubbard EM, Bernier R, Wiersema JR, Raymaekers R (2013). Developmental changes in mu suppression to observed and executed actions in autism spectrum disorders. Soc Cogn Affect Neurosci..

[113] Hobson HM, Bishop DVM (2016). Mu suppression – A good measure of the human mirror neuron system?. Cortex..

[114] Oberman LM, Ramachandran VS, Pineda JA (2008). Modulation of mu suppression in children with autism spectrum disorders in response to familiar or unfamiliar stimuli: the mirror neuron hypothesis. Neuropsychologia..

[115] Downing PE, Peelen MV (2011). The role of occipitotemporal body-selective regions in person perception. Cogn Neurosci..

[116] Grosbras M-H, Beaton S, Eickhoff SB (2012). Brain regions involved in human movement perception: a quantitative voxel-based meta-analysis. Hum Brain Mapp..

[117] Downing PE (2001). A cortical area selective for visual processing of the human body. Science (80- ).

[118] Noble K, Glowinski D, Murphy H, Jola C, McAleer P, Darshane N (2014). Event segmentation and biological motion perception in watching dance. Art Percept..

[119] Peelen MV, Downing PE (2007). The neural basis of visual body perception. Nat Rev Neurosci..

[120] Thompson JC, Baccus W (2012). Form and motion make independent contributions to the response to biological motion in occipitotemporal cortex. Neuroimage..

[121] Pelphrey KA, Shultz S, Hudac CM, Vander Wyk BC (2011). Research review: constraining heterogeneity: the social brain and its development in autism spectrum disorder. J Child Psychol Psychiatry..

[122] Peelen MV, Atkinson AP, Andersson F, Vuilleumier P (2007). Emotional modulation of body-selective visual areas. Soc Cogn Affect Neurosci..

[123] Han Z, Bi Y, Chen J, Chen Q, He Y, Caramazza A (2013). Distinct regions of right temporal cortex are associated with biological and human-agent motion: functional magnetic resonance imaging and neuropsychological evidence. J Neurosci..

[124] Matson JL, Neal D (2009). Diagnosing high incidence autism spectrum disorders in adults. Res Autism Spectr Disord..

[125] Valentine JC, Pigott TD, Rothstein HR (2010). How Many Studies Do You Need?. J Educ Behav Stat..

