# Correction to: Biological motion perception in autism spectrum disorder: a meta-analysis

**DOI:** 10.1186/s13229-021-00462-6

**Published:** 2021-08-20

**Authors:** Greta Krasimirova Todorova, Rosalind Elizabeth Mcbean Hatton, Frank Earl Pollick

**Affiliations:** 1 University of Glasgow, 62 Hillhead Street, Glasgow, G12 8AD UK; 2 Bradford District Care NHS Foundation Trust, Bradford, UK

## Correction to: Molecular Autism (2019) 10:49 https://doi.org/10.1186/s13229-019-0299-8

After publication of the original article [1] two errors were found:

1. Figure 4 Panel B shows green triangles indicating the wrong age group. The data points are accurate however, and the correct category of the data points can be easily inferred from Table 1
2. The authors re-evaluated the Cohen’s Kappa and PABAK of the first search in the meta-analysis. This is located in the Method section, at the end of the Study selection subsection. The authors discovered that after the update requested from the reviewers to contact some of the authors who did not provide statistics, we they did not re-calculate the Kappa and the PABAK accordingly. The differences in the actual numbers are minimal and they have no effect on the conclusions and interpretations of the paper.

The incorrect and correct figure 4 (Figs. 1 and 2 here) are shown in this correction article.

**Fig. 1:**
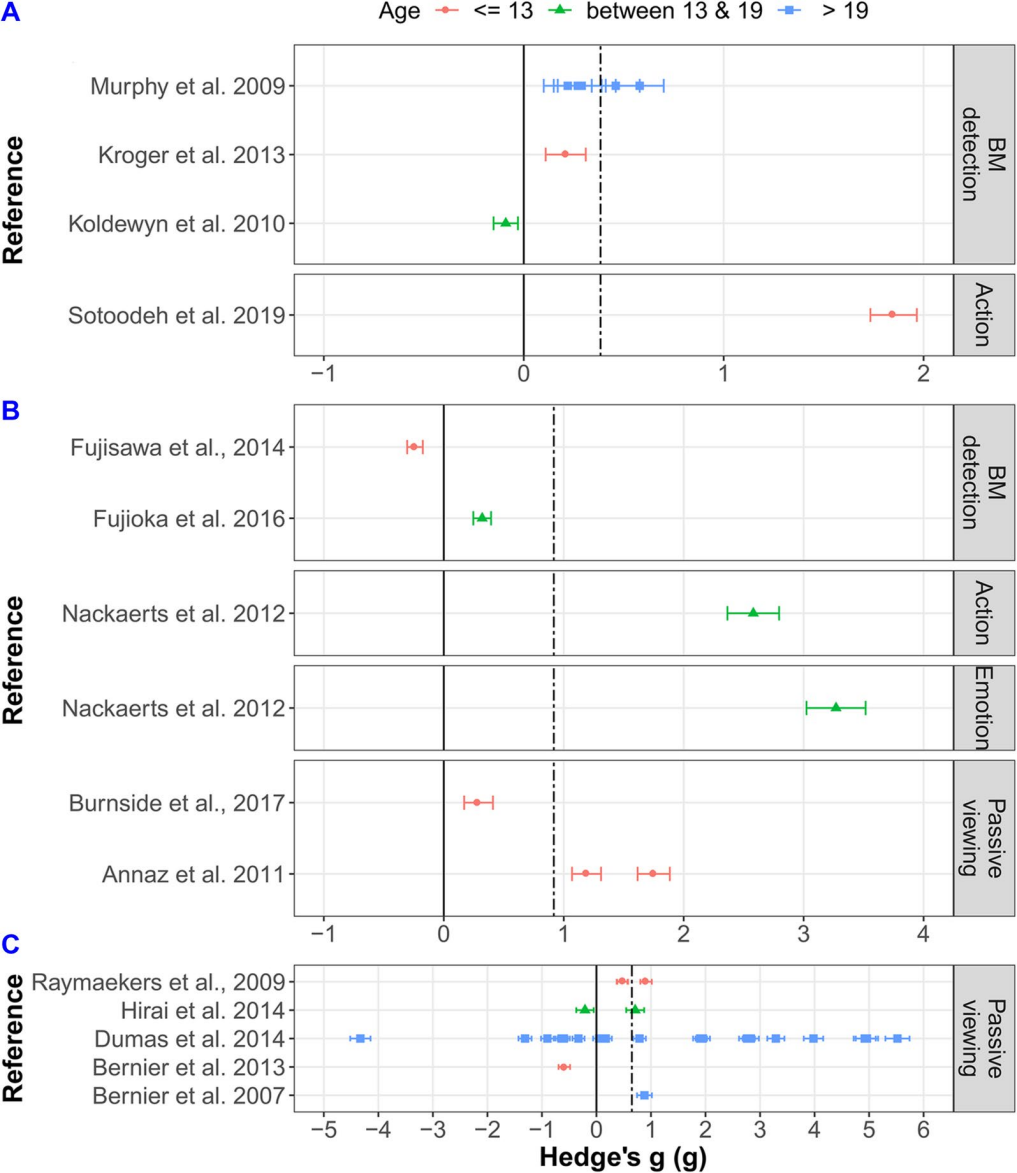
Incorrect figure 4

**Fig. 2:**
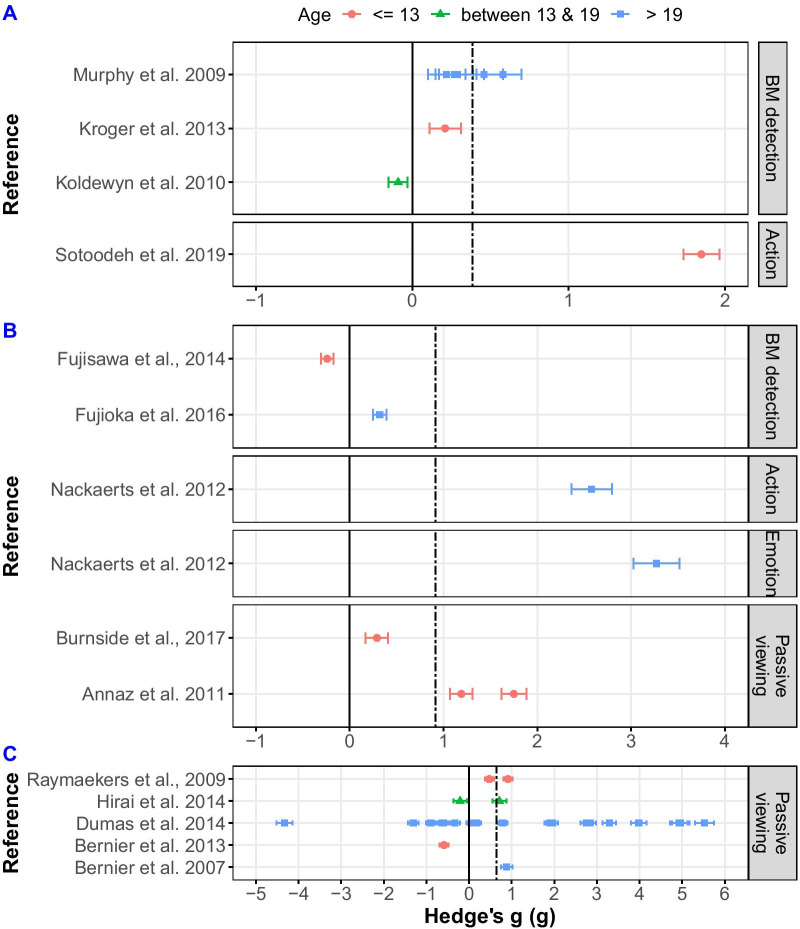
Correct figure 4

The updated values for the first search are as followed:

1. Cohen’s Kappa at the first search was calculated which equated to 64.07%. However, since Cohen’s Kappa is sensitive to distribution inequality [47] and ~ 92% of the records were classified as false positives, the prevalence index (0.816) and the prevalence-adjusted kappa (PABAK) of inter-rater reliability were calculated (PABAK = 87.98% inter-rater reliability, absolute agreement = 93.99%).

The original article has been updated.

## References

[1] Todorova GK, Hatton REM, Pollick FE (2019). Biological motion perception in autism spectrum disorder: a meta-analysis. Mol Autism.

