# Supplementary material for: Biological motion perception in autism spectrum disorder: a meta-analysis
Source: Mol Autism. 2019 Dec 18;10:49. doi: 10.1186/s13229-019-0299-8 (PMC6921539; doi:10.1186/s13229-019-0299-8)
Supplement: Supplementary file 1 — Additional file 1. Search strategies and number of records per database. File includes the search strategies used for the extraction of the papers from the five electronic databases mentioned in text and a table with the number of records extracted from each. [file 13229_2019_299_MOESM1_ESM.docx]

**Additional file 1**

Below are the search strategies used for the extraction of the papers form the five electronic databases.

**MEDLINE.** An example search strategy for MEDLINE® (1946 to November week 1 2017 (OVID) is shown below:

((autis* or asd or asperger* or "childhood schizophrenia" or kanner* or PDD-NOS or PDD* or "pervasive development* disorder*") and (PLD* or "biological motion" or "human motion" or "point-light display*" or "action observation*" or "action observation network*" or AON)).tw. limit 1 to english language

**WEB OF SCIENCE.** In Web of Science the following search strategy was used:

TS = (( autis* or asd or asperger* or "childhood schizophrenia" or kanner* or PDD-NOS or PDD* or "pervasive development* disorder*") AND ( PLD* or "biological motion" or "human motion" or "point-light display*" or "action observation*" or "action observation network*" or AON ))

Timespan: All years.

Search language=English

**PsycINFO.** The search strategy for PsycINFO (EBSCOhost) is shown below:

TX ( autis* or asd or asperger* or "childhood schizophrenia" or kanner* or PDD-NOS or PDD* or "pervasive development* disorder*") AND TX ( PLD* or "biological motion" or "human motion" or "point-light display*" or “action observation*” or “action observation network*” or AON ) TX (autis* or asd or asperger* or "childhood schizophrenia" or kanner) and (PLD* or "biological motion" or "human motion" or "point-light display*" or “action observation*” or “action observation network*” or AON) Limiters – English Search modes – Boolean/Phrase

**Dissertations & Theses A&I (ProQuest) and Dissertations & Theses: UK & Ireland (ProQuest).** The search strategy for Dissertations & Theses A&I (ProQuest) and Dissertations & Theses: UK & Ireland (ProQuest) is shown below.

all((autis* OR asd OR asperger* OR "childhood schizophrenia" OR kanner* OR "pervasive development* disorder*" OR PDD-NOS OR PDD*) ) AND all(("biological motion" OR "human motion" OR PLD* OR "point-light display*" OR "action observation*" OR "action observation network*" OR AON)) AND la.exact("English")

The number of extracted records from each database can be seen in the Table 1 below in descending order.

Table 1 Number of records extracted from each database.

| **Database** | **Records returned Search 1** | **Records returned Search 2** |
| --- | --- | --- |
| Web of Science | 483 | 102 |
| PsycINFO (EBSCOhost) | 163 | 1 |
| MEDLINE® (OVID) | 115 | 19 |
| Dissertations & Theses A&I (ProQuest) | 22 | 2 |
| Dissertations & Theses: UK & Ireland (ProQuest) | 10 | NA |
