# Supplementary material for: Biological motion perception in autism spectrum disorder: a meta-analysis
Source: Mol Autism. 2019 Dec 18;10:49. doi: 10.1186/s13229-019-0299-8 (PMC6921539; doi:10.1186/s13229-019-0299-8)
Supplement: Supplementary file 2 — Additional file 2. Weight of Evidence analysis. File includes the weight of evidence criteria for scoring and the summary statistics for Weight of Evidence scores. [file 13229_2019_299_MOESM2_ESM.docx]

**Additional file 2**

The strength of the body of evidence was assessed on both the study and review level using the Weight of Evidence approach developed by the EPPI-Centre (1) following the guidelines of Popay et al. (2). On the study level this includes the following four criteria on which each study will be judged (see Table 1 below):

1. Weight of evidence A (WOA)– Trustworthiness [taken from the quality assessment score, score obtained from quality assessment score ranges from 0 – 1: 0 – 0.333 scored as low, 0.334 – 0.666 scored as medium, 0.667 – 1 scored as high]; For fMRI papers an assessment was done using relevant criteria from the Standard Quality Assessment. Specifically questions related to analysis and results were excluded but the fMRI methodology was assessed for robustness. This was done collaboratively by the authors.
2. Weight of Evidence B (WOB) – appropriateness of the studies’ research design in terms of the current research question. This reflects the quality of the eligibility criteria. If the eligibility criteria were specific enough, then all studies should contribute to the interpretation of the body of evidence;
3. Weight of Evidence C (WOC) – This refers to the focus of the studies and whether their findings are generalizable to the question in hand;
4. Weight of Evidence D (WOD) – This refers to the overall score for each study, which is sum of the other three components. This is the score that is given to each study in Tables 1 and 2 in the main manuscript referred to as WoE (Weight of evidence).

Results for the overall weight of evidence and the sub-sections are shown below in Table 2.

Table 1 Weightings for Weight of Evidence C

| **Criteria** | **Weightings** | **Rationale** |
| --- | --- | --- |
| **Sample** | 3. (high) Fully describes sample (e.g. Diagnosis criteria (ADOS, ADI-R, clinical diagnosis, 3Di), Gender ratio, Age (mean, SD), FSIQ/VIQ/PIQ or other intelligence measures (mean and SD), Presence or absence of additional diagnosis was specified (TD and ASD), specifies the characteristics that TD individuals were matched to ASD individuals) | This will allow to determine how generalizable the findings are to a wider population of individuals on the autism spectrum |
|  | 2. (medium) Misses or not fully specifies one or two of the above listed sample characteristics (excluding diagnosis criteria as this was part of the eligibility criteria) |  |
|  | 1. (low) Misses more than two of the above listed sample characteristics |  |
| **Task** | 3. (high) Fully describes the paradigm used, the procedure and the participants’ task during the experiment; the procedure is randomised/counterbalanced | This will allow us to judge specific characteristics of the task and whether they could have contributed to any of the findings |
|  | 2. (medium) Elements about the paradigm are missing or participant’s task is not clearly stated. |  |
|  | 1. (low) The paradigm is poorly described, participants’ task is not specified |  |

Table 2 Weight of evidence average scores and standard deviation (SD) for each element.

|  | **WoA** | **WoB** | **WoC** | **WoD** |
| --- | --- | --- | --- | --- |
| **Average** | 2.963 | 3 | 2.454 | 8.417 |
| **SD** | 0.191 | 0 | 0.354 | 0.398 |

**References:**

1. Gough D. Weight of Evidence: a framework for the appraisal of the quality and relevance of evidence. Res Pap Educ. 2007 Jun;22(2):213–28.

2. Popay J, Roberts H, Sowden A, Arai L, Rodgers M, Britten N, et al. Guidance on the conduct of narrative synthesis in systematic reviews: A product from the ESRC Methods Programme. Lancaster University; 2006.
