# Supplementary material for: Biological motion perception in autism spectrum disorder: a meta-analysis
Source: Mol Autism. 2019 Dec 18;10:49. doi: 10.1186/s13229-019-0299-8 (PMC6921539; doi:10.1186/s13229-019-0299-8)
Supplement: Supplementary file 3 — Additional file 3. Exploratory EEG analysis. File includes an exploratory analysis of the EEG data with frequency as a predictive factor, as refered to from the text. [file 13229_2019_299_MOESM3_ESM.docx]

**Additional file 3**

An exploratory analysis was conducted on EEG studies to investigate the effect of the used frequency or measure to evaluate differences between ASD and NT individuals. The analysis showed that there was an overall effect of the type of frequency or measure used – *F*(3,25) = 3.22, *p* = 0.0397. If the frequency that was used was between 11-13 Hz, the estimated effect were very large – g = 2.452 [SE = 0.8054, 95% CIs: 0.7932 – 4.1108] *t*(25)=3.04, *p* = 0.0054], the effects were much smaller, but still large if the frequency was 8-13Hz g = 0.8822 [SE = 0.3871, 95% CIs: 0.0849 – 1.6794] *t*(25)=2.28, *p* < 0.0315]. If the frequency was 8-10Hz, the effect went in the opposite direction, with individuals with ASD showing larger but non-significant mu-suppression (g = -0.4031 [SE = 0.5185, 95% CIs: -1.4711 – 0.6648] *t*(25)=-0.78, *p* = 0.4442]). Finally, if N100 was measured, the effects were very small and non-significant (g = 0.2361 [SE = 0.9878, 95% CIs: -1.7984 – 2.2705] *t*(25)=-0.24, *p* = 0.8131]). It is note worthy that only one study looked at frequency 8-10Hz, one study looked at 11-13Hz and one study looked at N100. Thus, these results are unreliable, which can be seen from the large standard errors and confidence intervals. No further analyses were performed.
